# An Open Access Database of Licensed Cancer Drugs

**DOI:** 10.3389/fphar.2021.627574

**Published:** 2021-03-11

**Authors:** Pan Pantziarka, Rica Capistrano I, Arno De Potter, Liese Vandeborne, Gauthier Bouche

**Affiliations:** ^1^ The Anticancer Fund, Brussels, Belgium; ^2^ The George Pantziarka TP53 Trust, London, United Kingdom; ^3^ Faculty of Medicine, University of Leuven, Leuven, Belgium

**Keywords:** drug repurposing, drug licensing, antineoplastic drugs, licensed drugs, database, list of cancer drugs

## Abstract

A global, comprehensive and open access listing of approved anticancer drugs does not currently exist. Partial information is available from multiple sources, including regulatory authorities, national formularies and scientific agencies. Many such data sources include drugs used in oncology for supportive care, diagnostic or other non-antineoplastic uses. We describe a methodology to combine and cleanse relevant data from multiple sources to produce an open access database of drugs licensed specifically for therapeutic antineoplastic purposes. The resulting list is provided as an open access database, (http://www.redo-project.org/cancer-drugs-db/), so that it may be used by researchers as input for further research projects, for example literature-based text mining for drug repurposing.

## Introduction

The availability of curated datasets across diverse areas of medical research provides input to machine learning algorithms, natural language processing and the data mining of published scientific literature. In our own work on drug repurposing in oncology, our literature-based methodology relies heavily on database tables of drug repurposing candidates, molecular targets and pathways, the World Health Organization Essential Medicines List and so on (Pantziarka et al., 2018). A key input to this process is the list of drug candidates. For ‘hard repurposing’ projects, in which we are interested only in the potential of non-cancer drugs to be repurposed as cancer therapeutics, the ReDO_DB list provides a starting point for building a dataset for literature mining. But repurposing exists on a spectrum, with ‘soft’ repurposing representing the strategy of testing existing, licensed anticancer medications in cancers for which they are not currently approved (Pantziarka et al., 2020a). For a number of cancer-specific projects all forms of repurposing candidates, including cancer drugs that could be used outside of their licensed indications, needed to be included. In these cases we have not been able to identify a curated list of cancer drugs analogous to the ReDO list of repurposing candidates.

A similar issue arose in connection with automated searches of clinical trial registries to build datasets of clinical trials for manual assessment. In some cases these assessments are part of the process of identifying repurposing candidates for specific cancers, in other cases these searches are undertaken to support cancer patients looking for new therapeutic options. In both types of cases the unavailability of a curated list of licensed cancer drugs is an impediment.

Graph based methods are increasingly being used in the drug discovery process, including for repurposing (Gramatica et al., 2014). One recent example used such methods to search for new therapeutic avenues to explore in diffuse intrinsic pontine glioma (MacLean et al., 2020). Here too the availability of curated data enables rich networks to be developed in which multiple data sources can be used to construct models which embed implicit knowledge and new connections. Knowledge of which drugs are licensed as cancer medications can of course be discovered using such models. However, having a curated list reduces the need to do this and can lead to model simplification.

Given the need outlined above, a number of criteria for inclusion of a drug in the list emerge:

1.The drug is approved in the treatment of one or more malignancies by one or more regulatory agency (e.g. EMA or FDA)
2.The drug is used for anticancer effects rather than for supportive care, diagnostic use or for cancer-related co-morbidities

These simple criteria therefore exclude medicines commonly used in supportive care (analgesics, anti-emetics etc), and, more importantly, drugs which are in clinical development and which are therefore not approved. This includes drugs in late-stage development, including those in phase III randomized controlled trials.

There remain questions as to what constitutes a drug–is it the active pharmaceutical ingredient (API) or a specific drug product? How should different formulations of an API or drug be handled? Do products that include a number of anticancer APIs count as one or many drugs? Our starting point is that regulators grant approval to drug products rather than APIs. Secondly, we are interested in the generic names of the drugs rather than the commercial trade names which are used to differentiate competing products or which may be market-specific. In most, but not all, cases the generic drug name is the same as the API name. Therefore, we have adopted a pragmatic approach:

- 1.We are interested in licensed drug products rather than in commercial products–in other words cyclophosphamide or nivolumab rather than Cytoxan or Opdivo
- 2.Different pharmaceutical forms and strengths of the same drug (i.e., oral tablet or solution for intravenous injection) are not included as separate entries
- 3.Different commercial versions of the same drug (i.e., Doxil or Lipidox) are not included as separate entries
- 4.Different formulations of the same drug, for example paclitaxel and nab-paclitaxel, are counted as separate medicines as the licensing and approval for specific medical indications are different

For each drug entry we also list the anticancer APIs. For example paclitaxel and nab-paclitaxel are separate entries but they feature the same API. Other APIs included in a drug to alter bioavailability or enhance response, for example hyaluronidase in trastuzumab/hyaluronidase, are not listed with the anticancer APIs although they may be included in the drug product.

Here, we describe the methodology we have adopted to generate a new database of licensed anticancer drugs that we have published in an open access format online. We also highlight a number of existing data sources which are relevant to the problem we are solving, but which do not provide a complete solution to it.

## Methodology

An initial list of drugs used in oncology was created by extracting the list of cancer drugs from the NCI website (https://www.cancer.gov/about-cancer/treatment/drugs) on February 10, 2020, and combined with the informal lists we have previously developed for use in our repurposing projects. The NCI list contains drugs routinely used in cancer care for non-anti-neoplastic activity, for example anti-emetics for chemotherapy-induced nausea and vomiting. These drugs were filtered out to leave only the anticancer agents. A list of EMA-approved drugs was derived from the European Public Assessment Reports (EPARs) published on the EMA website (https://www.ema.europa.eu/en/medicines/download-medicine-data) on February 19, 2020. The list was filtered to remove non-oncology drugs and those not approved for human use. FDA approvals were cross-referenced with the Drugs@FDA website (https://www.accessdata.fda.gov/scripts/cder/daf/).

An additional source of drugs came from the World Health Organization (WHO) Anatomical Therapeutic Chemical (ATC) Classification System (https://www.whocc.no/). A downloadable version of the most recent list (August 3, 2020) is available for download at the Kyoto Encyclopedia of Genes and Genomes (KEGG) website (https://www.genome.jp/kegg-bin/get_htext?br08303.keg). All drugs in the ‘L Antineoplastic and Immunomodulating Agents’ class were extracted and cross-referenced with the EMA and NCI lists in order to identify and remove duplicates. The remaining drugs were manually assessed to identify those which are licensed anticancer medications and all others were excluded.

The drugs from these different sources were merged into a single list and duplicates identified and removed, along with any remaining non-antineoplastic agents. All drugs were identified by their International Non-proprietary Name (INN). For drugs lacking FDA or EMA approval we noted whether they were approved in Europe (from the period preceding the mandatory EMA centralized approval for cancer drugs) or elsewhere. In some cases this included ‘classical’ cytotoxic drugs such as doxorubicin and cyclophosphamide which are widely used in Europe but which were approved before the EMA central licensing process was instituted. These drugs were flagged as having European ‘national approval’. In a few other cases, such as the immunotherapeutic monoclonal antibody racotumomab, the drug is only approved in a few countries, in this case Cuba and Argentina. In such cases these approvals were listed explicitly after independent verification via the relevant regulatory agency website.

Finally, the anticancer API(s) of each drug was also identified, in the majority of cases this being the same as the drug INN. Each entry in the consolidated drug list was then cross-referenced with WHO Essential Medicines List (WHO EML) to identify those which are included in that list.

**FIGURE 1:**
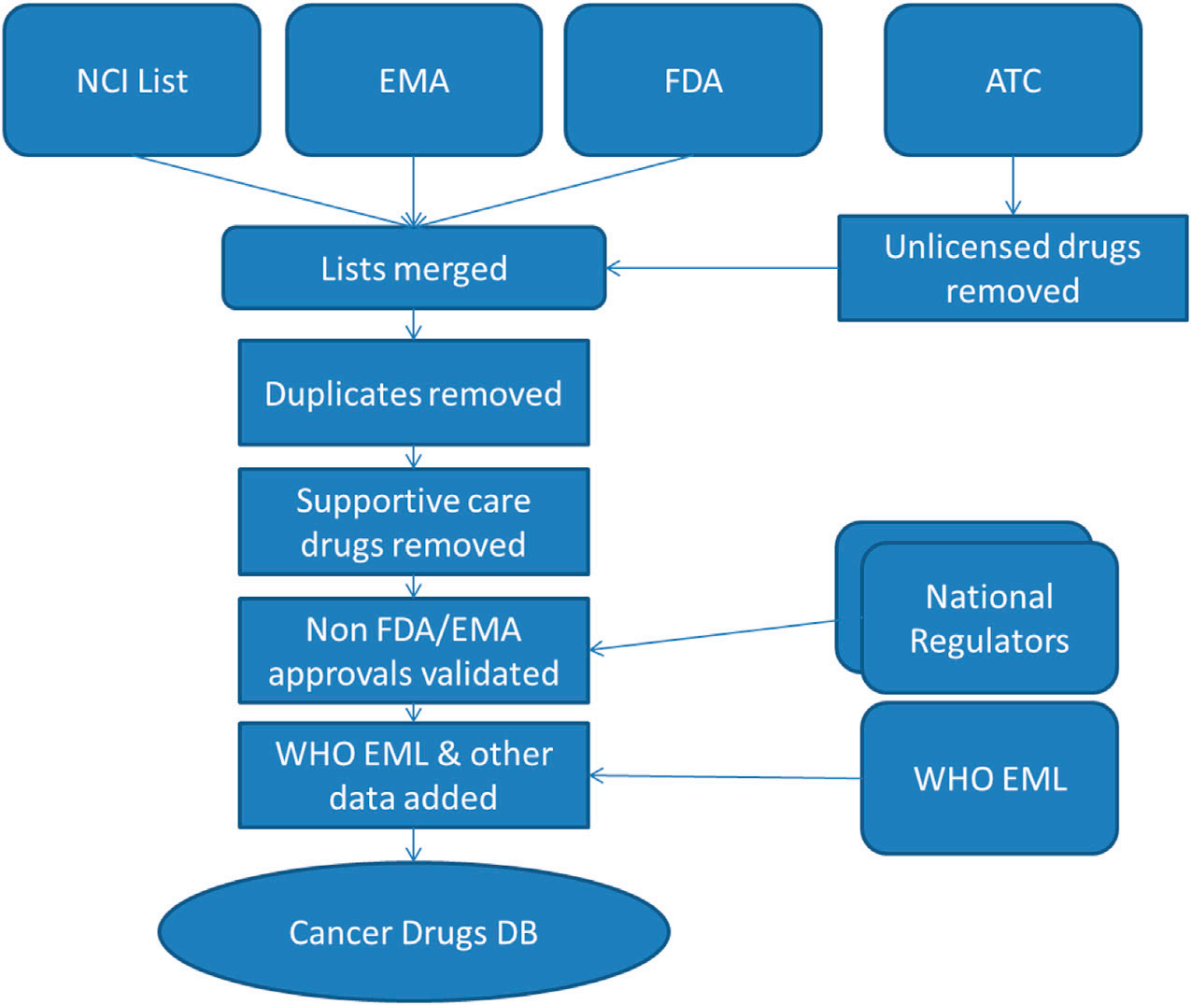
Database sources and build process. NCI, National *Cancer* Institute; EMA, European Medicines Agency; FDA, Food and Drug Administration; WHO EML, World Health Organization Essential Medicines List; DB, Database.

This process of data merging and cleansing, illustrated in Figure 1, has been performed iteratively and the list has been updated on December 21, 2020 to reflect recent drug approvals. A proactive approach has been adopted wherein monthly searches of relevant EMA and FDA publications listing drug approvals are manually scanned to identify new drugs which fit the criteria for inclusion in the database. It is also intended that there will be a twice-yearly scan to identify all drugs in the database which have been withdrawn.

Data on the medical uses for drugs is derived from the DrugBank database (Wishart et al., 2018). All medical indications are downloaded from DrugBank and filtered off-line to exclude non-cancer indications before being incorporated into the *Cancer* Drugs database. Drug target information is downloaded from the Drug-Gene Interaction database (DGIdb), which integrates target information from multiple sources, including DrugBank, the Drug Target Commons (Tang et al., 2018), PharmGKB (Thorn et al., 2013) and other databases (Cotto et al., 2017).

The patent status of a drug is a key consideration when selecting prospective repurposing candidates–for example in terms of support for clinical trials from manufacturers (Pantziarka et al., 2020b). A useful proxy for patent status is the year of first approval for a drug–a drug first approved in 1980, for example, is beyond the patent protection and drug exclusivity periods and is therefore unlikely to gain commercial sponsorship. Information on year of first approval is derived from a number of sources: KEGG (Kanehisa et al., 2010), DrugCentral (Avram et al., 2021) and the ChEMBL database (Cook et al., 2018). Where there are differences in first year approval date, for example a drug may be approved by FDA prior to EMA approval, the earliest approval year is used.

## Results

As of January 13, 2021 we have identified 313 drugs, of which 41 were approved for supportive care and two for cancer diagnostic purposes only. The total number of licensed anticancer drugs was 270, of which 243 (90%) were approved by the FDA, 168 (62%) had EMA approval and 50 (19%) were approved for use in different European countries through national approvals only. A further nine drugs (3%) had approvals in other parts of the world but no approval from the FDA, EMA or national agencies within Europe.

The vast majority of drugs, 268 (99%), have a single anticancer API, and only two drugs contain a combination of anticancer APIs.

Finally, 52 (19%) are included in the WHO EML.

The full list of drugs is shown in Supplementary Appendix.

**FIGURE 2:**
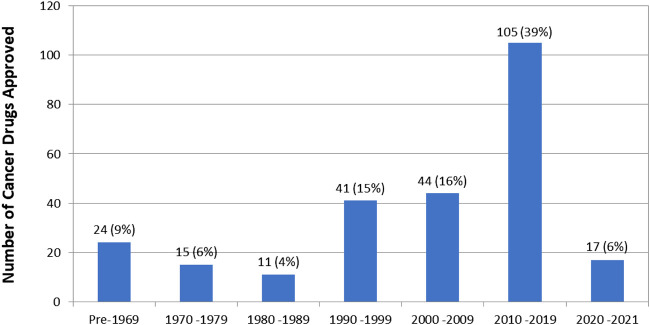
Number of drug approvals per decade. Note that this is the year of first approval of the drug regardless of indication. Some drugs, such as dexamethasone, were first approved for non-cancer indications.

First approval year data is available for 257 (95%) of the 270 cancer drugs. The number of drugs approved per decade is shown in Figure 2.

There is drug target data for 257 (95%) of cancer drugs, with the total number of unique drug targets being 963.

## Discussion

To our knowledge, we have generated the first comprehensive list of approved anticancer drugs. This list is available at http://www.redo-project.org/cancer-drugs-db/ and will be updated regularly to include new approvals and to exclude drugs that are no longer available. This open access list can be used as a source for multiple purposes, such as cancer drug meta-research or soft drug repurposing efforts.

This manually curated listing of anticancer drugs has a number of strengths and weaknesses. To our knowledge this is the only open access listing of medicines specifically approved for oncological use. While there are many partial listings of such drugs they are limited in that they have national or regional biases (e.g., NCI or EMA data), they often include drugs for diagnostics or supportive care and many include drugs which have yet to be approved (i.e., drugs still in development). Additionally, our process has removed duplicate entries and identified combination products.

An additional strength is that the listing is being published in an open access format, with a machine-readable download option to facilitate use of the data in further research work. For example, to facilitate the identification of new ‘soft repurposing’ opportunities which may have been previously over-looked.

There are a number of weaknesses also, the most obvious being the lack of information on drugs approved outside of Europe and the United States. While we have captured some of those instances (e.g. racotumomab approved in Cuba and *Argentina* or belotecan in South Korea), our methodology did not allow us to capture such cases systematically. This is particularly problematic for drugs only approved in countries with strong national drug development efforts. Japan has been a point in case for decades (Fujiwara, 1999), and more recently, and importantly for our study, the People’s Republic of China has also become very prominent (Wu et al., 2015). The National Medical Products Administration (NMPA), the Chinese drugs regulator, does not provide an English-language database or downloadable listing of approved medications. A number of drugs, for example the tyrosine kinase inhibitor icotinib, are reported by websites such as Wikipedia to have NMPA approval for cancer treatment, but without official verification this cannot be confirmed. According to the English-language version of the NMPA website a database is currently being implemented–when this is online the relevant data will be incorporated into our database.

A number of drugs which are known to be used off-label as cancer treatments have not been included. Zoledronic acid, for example, is used on-licence to prevent bone-related events in advanced cancer and is therefore classed as a drug for supportive care in our database. However, it is also used off-label to prevent cancer recurrence in breast cancer (Ottewell and Wilson, 2019). If the license is updated to include this new medical use then zoledronic acid would be included as an anticancer drug.

In addition to maintaining the currency of the data through periodic monitoring of new drug approvals, we intend to enhance and expand the range of data collected for each entry in the database.

## Data Availability

The datasets presented in this study can be found in online repositories. The names of the repository/repositories and accession number(s) can be found below: https://osf.io/nk8pg/ http://www.redo-project.org/cancer-drugs-db/.
