# Supplementary material for: An Open Access Database of Licensed Cancer Drugs
Source: Front Pharmacol. 2021 Mar 11;12:627574. doi: 10.3389/fphar.2021.627574 (PMC7991999; doi:10.3389/fphar.2021.627574)
Supplement: Supplementary file 1 [file table1.docx]

## Appendix 1

The Cancer Drugs database as of 13/01/21.

| Product | EMA | FDA | EN | Other | WHO | Year | Indications | Targets |
| --- | --- | --- | --- | --- | --- | --- | --- | --- |
| Abemaciclib | Y | Y | N |  | N | 2017 | Advanced Breast Cancer, Metastatic Breast Cancer | CDK6, CDK4, CCND3, CDKN2A, CCND2, SMARCA4 |
| Abiraterone | Y | Y | N |  | Y | 2011 | Metastatic Castration Resistant Prostate Cancer | CYP17A1, DSE, TSPYL1 |
| Acalabrutinib | Y | Y | N |  | N | 2017 | Chronic Lymphocytic Leukaemia (CLL), Mantle Cell Lymphoma (MCL), Small Lymphocytic Lymphoma | BTK, TEC, TXK, BMX |
| Aclarubicin | N | N | N | Japan | N |  |  | SMN1, TOP2B, CSF2 |
| Afatinib | Y | Y | N |  | N | 2013 | Metastatic Non-Small Cell Lung Cancer, Refractory, metastatic squamous cell Non-small cell lung cancer | EGFR, ERBB2, ERBB4, KRAS, EML4, NRG1, NRAS, ERBB3 |
| Aflibercept | Y | Y | N |  | N | 2011 | Metastatic Colorectal Cancer (MCRC) | VEGFA, VEGFB, PGF, PIGF, VEGFC |
| Aldesleukin | N | Y | Y |  | N | 1992 | High Risk Neuroblastoma, Metastatic Melanoma, Metastatic Renal Cell Carcinoma | IL2RG, IL2RB, IL2RA, IL2 |
| Alectinib | Y | Y | N |  | N | 2015 | Refractory, metastatic Non small cell lung cancer | ALK, EML4, NPM1, CDKN2A, SMAD4, RET |
| Alemtuzumab | Y | Y | N |  | N | 2001 | B-Cell Chronic Lymphocytic Leukemia, T-Cell Prolymphocytic Leukemia | CD52, FCGR3B, FCGR2A, FCGR2B, FCGR3A, FCGR1A, FCGR2C, CXCL12 |
| Alitretinoin | Y | Y | N |  | N | 1999 |  | RXRG, VKORC1, RARA, RXRA, RXRB, RARB, RARG, CYP26C1, IGFBP3, PSG5 |
| Alpelisib | Y | Y | N |  | N | 2019 | Advanced Metastatic Breast Cancer | PIK3R4, PIK3R6, PIK3CA, SGK1, PI4KA |
| Altretamine | N | N | Y |  | N | 1990 | Ovarian Cancer, Ovarian Cancer Stage III |  |
| Aminolevulinic Acid | Y | Y | N |  | N | 1999 | Basal Cell Carcinoma (BCC), Squamous Cell Carcinoma (SCC) |  |
| Amrubicin | N | N | N | Japan | N | 2002 |  | TOP2A |
| Amsacrine | N | N | N |  | N | 1987 | Leukemia, Acute, Refractory Leukemia | TOP2A, ALB, ORM1, BTF3P11 |
| Anastrozole | N | Y | Y |  | Y | 1995 | Advanced Breast Cancer, Early Breast Cancer, Locally Advanced Breast Cancer (LABC), Metastatic Breast Cancer, Invasive, early Breast Cancer | CYP19A1, TNFRSF11B, UGT1A4, CYP1B1, IGF2, KMT2C, TNFSF11 |
| Apalutamide | Y | Y | N |  | N | 2018 | Nonmetastatic Prostate Cancer | AR, GABRA1 |
| Arsenic Trioxide | Y | Y | N |  | Y | 2018 | Refractory Acute Promyelocytic Leukemia | TXNRD1, TXNRD2 |
| Asparaginase | Y | Y | N |  | Y | 1994 | Acute Lymphoblastic Leukaemias (ALL) | CPA2, NOTCH1, LPL, ETV6, ASNS, TYMSOS, GATA3, HLA-DRB1, PYGL, GRIA1, NFATC2, PNPLA3, SOD2, RB1 |
| Atezolizumab | Y | Y | N |  | N | 2016 | Metastatic Non-Small Cell Lung Cancer, Small Cell Lung Cancer (SCLC), Triple Negative Breast Cancer (TNBC), Urothelial carcinoma ureter metastatic, Locally advanced Urothelial Carcinoma | CD274, ARID1A, CTLA4, MDM4, ALK, STK11, HLA-DRA, CX3CL1, PBRM1 |
| Avapritinib | Y | Y | N |  | N | 2020 | Metastatic Gastrointestinal Stromal Tumor, Unresectable Gastrointestinal stromal tumor | KIT, PDGFRA |
| Avelumab | Y | Y | N |  | N | 2017 | Locally advanced disease has progressed during or following platinum-containing chemotherapy urothelial carcinoma (UC), Locally advanced disease has progressed within 12 months of neoadjuvant or adjuvant treatment with platinum-containing chemotherapy urothelial carcinoma (UC), Metastatic Merkel Cell Carcinoma (MCC), Metastatic disease has progressed during or following platinum-containing chemotherapy urothelial carcinoma (UC), Metastatic disease has progressed within 12 months of neoadjuvant or adjuvant treatment with platinum-containing chemotherapy urothelial carcinoma (UC) | CD274, EGFR |
| Axicabtagene Ciloleucel | Y | Y | N |  | N | 2017 | Refractory Diffuse large B-cell lymphoma NOS, Refractory High grade B-cell lymphoma Burkitt-like lymphoma, Refractory Primary Mediastinal Large B-Cell Lymphoma, Relapsed Diffuse large B-cell lymphoma NOS, Relapsed High grade B-cell lymphoma Burkitt-like lymphoma, Relapsed Primary Mediastinal Large B-Cell Lymphoma |  |
| Axitinib | Y | Y | N |  | N | 2012 | Advanced Thyroid cancer | FLT1, KDR, PDGFB, KIT, FLT4, ABL1, VHL, UGT1A1 |
| Azacitidine | Y | Y | N |  | N | 2004 | Acute Myeloid Leukemia (AML), Chronic Myelomonocytic Leukemia | DNMT1, DNMT3A, CD247, AFP, ASXL1, IL11, TAGLN, LIF, TRIT1, GATA2, CDA, TET2 |
| BCG Vaccine | Y | Y | N |  | Y |  | Recurrent Superficial Bladder Cancer, Carcinoma in situ of urinary bladder |  |
| Belantamab Mafodotin | Y | Y | N |  | N | 2020 | Relapsed Or Refractory Multiple Myeloma |  |
| Belinostat | N | Y | N |  | N | 2014 | Relapsed Peripheral T-Cell Lymphoma, Refractory Peripheral T-cell Lymphoma Unspecified | HDAC2, HDAC5, HDAC4, HDAC10, HDAC7, HDAC11, HDAC9, HDAC1, HDAC3, HDAC6, HDAC8, KMT2A, UGT1A1, FBXW7 |
| Belotecan | N | N | N | South Korea | N | 2003 |  |  |
| Bendamustine | N | Y | Y |  | Y | 2008 | Chronic Lymphocytic Leukaemia (CLL), Follicular Non-Hodgkin&#39;s Lymphoma Refractory, Refractory Hodgkin Lymphoma, Refractory Mantle Cell Lymphoma, Waldenstrom&#39;s Macroglobulinemia (WM), Recurrent multiple myeloma, Refractory indolent B cell non-hodgkin lymphoma | CD69, ATM |
| Bevacizumab | Y | Y | N |  | Y | 2004 | Cervical Cancer Metastatic, Metastatic Colorectal Cancer (MCRC), Metastatic Non-Squamous Non-Small Cell Lung Cancer, Metastatic Renal Cell Carcinoma, Persistent Cervical Cancer, Recurrent Cervical Cancer, Recurrent Glioblastoma, Stage III epithelial ovarian cancer following initial surgical resection, Stage IV epithelial ovarian cancer following initial surgical resection, Fallopian tube cancer following initial surgical resection, Locally advanced nonsquamous non-small cell lung cancer, Primary peritoneal cancer following initial surgical resection, Recurrent Non-Squamous Non-Small Cell Lung Cancer, Recurrent Platinum-Sensitive Epithelial Ovarian Cancer, Recurrent Platinum-resistant Epithelial Ovarian Cancer, Recurrent platinum drug resistant Fallopian tube cancer, Recurrent platinum drug resistant primary peritoneal cancer, Recurrent platinum sensitive primary peritoneal cancer, Recurrent platinum-sensitive fallopian tube cancer, Unresectable Non-Squamous Non-Small-Cell Lung Cancer | VEGFA, EDN1, VEGFC, MGAT4A, FCGR2B, VEGFB, FCGR1A, C1QA, RGS5, NF1, THBS2, ARMS2, SHMT1, FCGR2C, C1QB, CFH, GGH, MTHFR, VHL, GC, FCGR2A, NF2, C1QC, HTRA1 |
| Bexarotene | Y | Y | N |  | N | 1999 | Refractory peripheral cutaneous T-cell lymphoma | RXRG, RXRA, RXRB, NR1H3, MAPK8 |
| Bicalutamide | N | Y | Y |  | Y | 1995 | Stage D2 Prostatic carcinoma | CDH1, KMT2D, CFLAR, BAX, CYP2B6, FST, KLK3 |
| Binimetinib | Y | Y | N |  | N | 2018 | Metastatic Melanoma, Unresectable Melanoma | MAP2K1, MAP2K3, MAP2K2, MAP2K5, MAP2K6, MAP2K7, MAP2K4, MAP3K1, BRAF, NF1, KRAS, APC, GNAQ, NRAS, GNA11, HRAS |
| Bleomycin | N | Y | Y |  | Y | 1973 | Cervical Cancers, Head and Neck Carcinoma, Intracranial Germ Cell Tumors, Lymphoma, Hodgkins, Malignant Pleural Effusions, Non-Hodgkin&#39;s Lymphoma (NHL), Ovarian Cancer, Penile Cancer, Testicular Cancer, Vulvar Cancers, Early Kaposi&#39;s sarcoma | LIG3, LIG1, F2R, CTLA4, GJA1, AFP, ADK, ERCC2, GSTM1, CCL17, SMAD2, IFNG |
| Blinatumomab | Y | Y | N |  | N | 2014 | Precursor B-Lymphoblastic Lymphoma/Leukaemia Refractory, Relapsed B cell precursor Acute lymphoblastic leukemia | CD19, CD3E, CD3D, BCR, ABL1, CD247, CD3G |
| Bortezomib | Y | Y | N |  | Y | 2003 | Mantle Cell Lymphoma (MCL), Multiple Myeloma (MM) | PSMC2, PSMD4P1, PSMD11, PSMB3, PSMD3, PSMA8, PSMB10, PSMC3IP, PSMD14, PSMD8, PSMD13, PSMC1P1, PSMD10P2, PSME3, PSMB1, PSMD9, PSMD10P3, PSMD6, PSMD12, PSMC3, PSME2P2, PSMA1, PSMD4, PSMC4, PSMB8, PSMD2, PSME1, PSMD10, PSMD5, PSMA6, PSMD1, PSMA3, PSME2, PSMA4, PSMC5, PSMC1, PSMB6, PSMB5, PSMB4, PSMB9, PSME4, PSMA2, PSMB7, PSMD7, PSMB2, PSMB11, PSMA7, ADRM1, PSMA5, PSMF1, PSMC6, PIPSL, PSMD10P1, SEM1, BCL2L2, PMAIP1, KLK4, PRSS3, F12, GATA2, CAPN1, CTSL |
| Bosutinib | Y | Y | N |  | N | 2010 | Refractory, accelerated phase Chronic myelogenous leukemia, Refractory, blast phase Chronic myelogenous leukemia, Refractory, chronic phase Chronic myelogenous leukemia | TXK, HCK, SIK2, ABL1, SRC, MAP3K2, SIK3, CAMK2G, STK4, CAMK1D, SIK1, BCR, STK24, STK10 |
| Brentuximab vedotin | Y | Y | N |  | N | 2011 | B-large cell anaplastic Lymphoma (Kiel Classification) refractory, Refractory Hodgkin Lymphoma, Post-autologous hematopoietic stem cell transplantation Hodgkin lymphoma | TNFRSF8 |
| Brexucabtagene Autoleucel | Y | Y | N |  | N | 2020 | Refractory Mantle Cell Lymphoma, Relapsed Mantle Cell Lymphoma |  |
| Brigatinib | Y | Y | N |  | N | 2017 | Metastatic Non-Small Cell Lung Cancer | IGF1R, ALK, EGFR, ABL1, ROS1 |
| Buserelin | N | N | Y |  | N | 2017 | Stage D2 Prostatic carcinoma | GNRHR, LHCGR, IGFBP1 |
| Busulfan | Y | Y | N |  | N | 1954 | Essential Thrombocythemia (ET), Polycythemia Vera (PV), Chronic Chronic myelogenous leukemia | GSTA1, BCR, GSTM1, GALC, ITGAL, CTH, ADK |
| Cabazitaxel | Y | Y | N |  | N | 2010 | Refractory, metastatic hormone-refractory Prostate cancer | TUBB1, TUBA4A, IGF2, TUB |
| Cabozantinib | Y | Y | N |  | N | 2012 | Advanced Renal Cell Carcinoma, Hepatocellular Carcinoma, Metastatic Clear Cell Renal Cell Carcinoma, Progressive, metastatic Medullary thyroid cancer | RET, KDR, MET, KIT, GNA11, CFLAR, HGF, ROS1 |
| Calaspargase Pegol | N | Y | N |  | N | 2018 | Acute Lymphoblastic Leukaemias (ALL) |  |
| Capecitabine | Y | Y | N |  | Y | 1998 | Duke&#39;s C Colon cancer, Esophageal Cancers, Hepatobiliary Cancers, Malignant Neoplasm of Stomach, Metastatic Breast Cancer, Metastatic Colorectal Carcinoma, Pancreatic Cancer Metastatic, Metastatic pancreatic endocrine carcinoma, Refractory Ovarian cancer, Refractory falopian tube cancer, Refractory peritoneal cancer, Refractory, metastatic Colorectal carcinoma | TYMS, MGAT4A, DLG5, CDH1, DPYD, MTHFR, UMPS, EXO1, AREG, MIR2054, MIR27A, ADCY2, CES1P1, TYMP, CDA, SELE, ENOSF1, HLA-G, TYMSOS, SLC22A7, REV3L |
| Capmatinib | N | Y | N |  | N | 2020 | Metastatic Non-Small Cell Lung Cancer | MET, BRAF |
| Carboplatin | N | Y | Y |  | Y | 1989 | Advanced Cervical Cancer, Advanced Endometrial Cancer, Advanced Esophageal Cancers, Advanced Head and Neck Cancer, Advanced Melanoma, Advanced Non Small Cell Lung Cancer, Advanced Ovarian Carcinoma, Advanced Sarcoma, Metastatic Breast Cancer, Neuroendocrine Carcinoma of the Skin, Pleural mesothelioma malignant, Refractory Hodgkin Lymphoma, Retinoblastoma, Advanced Bladder cancer, Advanced Small cell lung cancer, Advanced Testicular cancer, Advanced Thymoma, Advanced thymic carcinoma, Refractory Non-Hodgkin&#39;s lymphoma | GCLC, ATP7A, GPX5, PRRC2A, TP73, SOX10, C6ORF15, EIF3A, ERCC1, DOCK8, DSCAM, MSH5, SLC31A1, TRIM5, MAD1L1, ATP7B, ATAT1, SERPINA5, ETS2, DPCR1, XYLT2, PSORS1C1, CDSN, MTR, BARD1, RNF8, PPP1R18, HCP5 |
| Carfilzomib | Y | Y | N |  | N | 2009 | Refractory Multiple Myeloma, Waldenstrom&#39;s Macroglobulinemia (WM) | PSMB2, PSMD14, PSMB8, PSMD13, PSMC6, PSMC5, PSMA6, PSMD6, PSMC1, PSMA5, PSME2, PSMA2, PSMB5, PSMA3, PSMD10P2, PSMB9, PSMD3, PSMB7, PIPSL, PSMA7, PSMB4, PSMD4, SEM1, PSMC3, PSMB6, PSMD2, PSMD7, PSMD12, PSMC4, PSMB10, PSMA1, PSMD5, PSMD8, PSMC2, PSMB11, PSMA4, PSMD11, PSMD1, PSMB1, ADRM1, PSMA8, PSMB3, PSME1, PSMD10P3, PSME2P2, PSMD4P1, PSMC1P1, PSME4, PSME3, PSMD10, PSMD10P1, PSMF1, PSMD9, PSMC3IP, PRB2 |
| Carmofur | N | N | N | Japan | N |  |  |  |
| Carmustine | Y | Y | N |  | N | 1977 | Astrocytomas, Brain Stem Gliomas, Ependymomas, Glioblastomas, Medulloblastomas, Mycosis Fungoides (MF), Recurrent Glioblastoma Multiforme, Refractory Hodgkin Lymphoma, Refractory Multiple Myeloma, Tumors Metastatic to Brain, High grade newly diagnosed Glioma, Refractory Non-Hodgkin&#39;s lymphoma | GSR, MGMT, PLAU, E2F1 |
| Carmustine Implant | Y | Y | N |  | N | 1977 | Astrocytomas, Brain Stem Gliomas, Ependymomas, Glioblastomas, Medulloblastomas, Mycosis Fungoides (MF), Recurrent Glioblastoma Multiforme, Refractory Hodgkin Lymphoma, Refractory Multiple Myeloma, Tumors Metastatic to Brain, High grade newly diagnosed Glioma, Refractory Non-Hodgkin&#39;s lymphoma | GSR, MGMT, PLAU, E2F1 |
| Cemiplimab | Y | Y | N |  | N | 2018 | Metastatic cutaneous squamous cell carcinoma, Locally advanced cutaneous squamous cell carcinoma, and not a candidate for curative surgery or curative radiation | PDCD1, CDK12 |
| Ceritinib | Y | Y | N |  | N | 2014 | Refractory, locally advanced Non-small cell lung cancer, Refractory, metastatic Non small cell lung cancer | IGF1R, ALK, INSR, TSSK1B, EML4, ROS1, NPM1 |
| Cetuximab | Y | Y | N |  | N | 2004 | Metastatic Colorectal Cancer (MCRC), Metastatic Squamous Cell Carcinoma of the Head and Neck, Advanced squamous cell carcinoma of the head and neck | EGFR, CCND1, MGAT4A, NRAS, C1QC, FCGR3A, HBEGF, NRG2, BTC, FCGR2A, AREG, DNTT, BRAF, C1QB, NRG3, EGF, GC, TGFA, C1QA, VPS37A, EREG, EPO, KRAS, PTP4A3 |
| Chlorambucil | N | Y | Y |  | Y | 1957 | Chronic Lymphocytic Leukaemia (CLL), Hodgkins Disease (HD), Non-Hodgkin&#39;s Lymphoma (NHL), Waldenstrom&#39;s Macroglobulinemia (WM), Giant follicular lymphoma | MGMT, CXCL12, GSTM1, BRCA2, FANCC |
| Chlormethine | Y | Y | N |  | N | 1949 | Carcinoma, Bronchogenic, Chronic Lymphocytic Leukaemia (CLL), Hodgkins Disease (HD), Lymphoma, Diffuse, Mycosis Fungoides (MF), Polycythemia Vera (PV), Stage I Mycosis Fungoides, Malignant effusion | CSF2 |
| Cisplatin | N | Y | Y |  | Y | 1978 | Advanced Pancreatic Cancer, Breast Cancer, Cervical Cancers, Esophageal Cancers, Head and Neck Carcinoma, Hepatobiliary Cancers, Hepatoblastomas, Malignant Neoplasm of Stomach, Medulloblastomas, Metastatic Melanoma, Multiple Myeloma (MM), Neuroblastomas, Non-Small Cell Lung Carcinoma (NSCLC), Ovarian Cancer Metastatic, Pleural Mesotheliomas, Primary Central Nervous System Lymphoma (PCNSL), Recurrent Endometrial Cancer, Refractory Hodgkin Lymphoma, Sarcoma, Osteogenic, Small Cell Lung Cancer (SCLC), Thymic Cancer, Advanced Bladder cancer, Metastatic Anal cancer, Metastatic Endometrial cancer, Metastatic Penile cancer, Metastatic Testicular cancer, Refractory Non-Hodgkin&#39;s lymphoma | ERCC2, GADD45A, ACYP2, SLC22A2, CSNK2A3, RARS, FANCA, UBE2I, LRP2, LIG3, REV1, ERCC1, MIR21, OTOS, KLC3, DRAM1, BIRC7, DCBLD1, GALNT18, XPC, GABPA, MT1F, DFFB, ATP7B, GALNT14, MUTYH, ERCC4, EIF3A, ATOX1, MLLT3, KRT20, MPG, TERC, MT1H, SLC31A1, CDH17, FANCC, SLC16A5 |
| Cladribine | Y | Y | N |  | N | 1993 | Chronic Lymphocytic Leukaemia (CLL), Cutaneous T-Cell Lymphoma (CTCL), Hairy Cell Leukemia (HCL), Non-Hodgkin&#39;s Lymphoma (NHL) | RRM2B, RRM1, POLE4, RRM2, POLE2, POLE3, POLE, POLA1, PNP, DIABLO, NT5C1A, XIAP, DCK, BCL2L2, CDKN1A |
| Clofarabine | Y | Y | N |  | N | 2004 | Acute Lymphoblastic Leukaemia Recurrent, Refractory Acute Lymphoblastic Leukemia, Refractory Acute Myelogenous Leukemia (AML), Refractory Langerhans cell histiocytosis | POLA1, RRM1, RRM2, POLE, RRM2B, POLD1, APBB1 |
| Cobimetinib | Y | Y | N |  | N | 2015 | Metastatic Melanoma, Unresectable Melanoma | MAP2K1, MAP2K7, MAP2K2, MAP2K6, MAP2K3, MAP2K4, MAP2K5, RAF1, ARAF, BRAF, NF1 |
| Copanlisib | N | Y | N |  | N | 2017 | Relapsed Follicular Lymphoma | PIK3C2B, PIK3CD, PIK3C2A, PIK3R6, PIK3R3, PIK3CA, PIK3R1, PIK3R2, PIK3R5, PIK3R4 |
| Crizotinib | Y | Y | N |  | N | 2011 | Metastatic Non-Small Cell Lung Cancer | MET, ROS1, ALK, EML4, NPM1, ETV6, PTPN12, SMAD4, AREG, CDKN2A |
| Cyclophosphamide | N | Y | Y |  | Y | 1959 | Acute Lymphoblastic Leukaemias (ALL), Acute Myelocytic Leukemia, Adenocarcinoma of the Ovaries, Breast Cancer, Burkitt&#39;s Lymphoma, Chronic Lymphocytic Leukaemia (CLL), Chronic Myeloid Leukemia (CML), Disseminated Neuroblastoma, Ewing&#39;s Sarcoma, Lung Cancers, Lymphoma, Hodgkins, Multiple Myeloma (MM), Non-Hodgkin&#39;s Lymphoma (NHL), Ovarian germ cell tumour, Pheochromocytomas, Retinoblastoma, Rhabdomyosarcomas, Waldenstrom&#39;s Macroglobulinemia (WM), Advanced Alibert-Bazin syndrome, Histiocytic lymphoma, Metastatic gestational trophoblastic tumor, Mixed-cell type lymphoma, Refractory Small cell lung cancer, Relapsed Wilm&#39;s tumor | SLC22A16, GNL3, XRCC1, CYP2B6, TP53AIP1, PNPLA3, DROSHA, MUTYH, LIG3, PERP, ACTB, PPP2R5D, CCNK, EPHX1, TNFRSF1A, GSTA1, FOXO1, CTH |
| Cytarabine | Y | Y | N |  | Y | 1969 | Acute Lymphocytic Leukemia (ALL), Acute Myeloid Leukemia (AML), Acute Promyelocytic Leukemia (APL), Leptomeningeal Metastases, Meningeal leukemia, Non-Hodgkin&#39;s Lymphoma (NHL), Blast phase Chronic myelocytic leukemia | POLE, POLD1, SLC22A12, DROSHA, RRM2B, GATA1, GATA3, IKZF1, NT5C3A, WT1, DCK, MED12L, LINC00251, CDA, DOK5, BMP7, SULT2B1 |
| Dabrafenib | Y | Y | N |  | N | 2013 | Metastatic Melanoma, Unresectable Melanoma | SIK1, BRAF, NEK11, CDKN2A, STAG2, NRAS, ATXN1L, EZH2, RAC1, MAP2K1, NF1 |
| Dacarbazine | N | Y | Y |  | Y | 1975 | Advanced Soft Tissue Sarcoma, Lymphoma, Hodgkins, Metastatic Melanoma, Pheochromocytomas, Advanced Medullary thyroid cancer, Advanced Pancreatic neuroendocrine tumor | PGD, POLA2, ADK, MGMT, CYP1A1, GSTM1 |
| Dacomitinib | Y | Y | N |  | N | 2018 | Metastatic Non-Small Cell Lung Cancer | EGFR, ERBB3, ERBB4, ERBB2, PTEN |
| Dactinomycin | N | Y | N |  | Y | 1964 | Ewing&#39;s Sarcoma, Ovarian Cancer, Rhabdomyosarcomas, Sarcoma, Osteogenic, Wilms&#39; tumor, Metastatic nonseminomatous Testicular cancer |  |
| Daratumumab | Y | Y | N |  | N | 2015 | Multiple Myeloma (MM), Refractory Multiple Myeloma, Relapsed Multiple Myeloma, Relapsed Or Refractory Multiple Myeloma | CD38 |
| Daratumumab, Hyaluronidase | N | Y | N |  | N | 2015 | Multiple Myeloma (MM), Refractory Multiple Myeloma, Relapsed Multiple Myeloma, Relapsed Or Refractory Multiple Myeloma | CD38 |
| Darolutamide | Y | Y | N |  | N | 2019 | Non-mestatatic castrate-resistant prostate cancer | AR, PGR |
| Dasatinib | Y | Y | N |  | Y | 2006 | Acute Lymphoblastic Leukaemias (ALL), Chronic Myeloid Leukemia (CML) | ABL2, KIT, EPHA2, FGR, ABL1, BCR, SIK3, STAT5B, DDR2, ABI1, TEC, NR4A3, CRKL, EPHA5, ARID1A, PDGFRA, CBL, CSK, IDH2, PPAT |
| Daunorubicin | N | Y | Y |  | Y | 1979 | Acute Lymphocytic Leukemia (ALL), Acute Myeloid Leukemia (AML), Acute erythroid leukemia, Acute monocytic leukaemia | ATP7B, SETD4, GATA1, RPSA, NRP2, FMO3, WT1, DROSHA, CBR3, IKZF1, PNPLA3, AKR1C4, AKR7A2 |
| Daunorubicin, Cytarabine Liposome | Y | Y | N |  | N |  | Acute Lymphocytic Leukemia (ALL), Acute Myeloid Leukemia (AML), Acute erythroid leukemia, Acute monocytic leukaemia |  |
| Decitabine | Y | Y | N |  | N | 2006 | Chronic Myelomonocytic Leukemia, Myelodysplastic Syndromes (MDS) | DNMT1, DNMT3A, ASXL1, GSTP1, IL2RA, MUC2, TET2 |
| Decitabine, Cedazuridine | N | Y | N |  | N | 2006 | Chronic Myelomonocytic Leukemia, Myelodysplastic Syndromes (MDS) | DNMT1, DNMT3A, ASXL1, GSTP1, IL2RA, MUC2, TET2 |
| Degarelix | Y | Y | N |  | N | 2008 | Advanced Prostate Cancer | GNRHR, GNRHR2 |
| Denileukin Diftitox | N | Y | N |  | N | 1999 | Cutaneous T-Cell Lymphoma (CTCL) | EEF2, IL2RB, IL2RA, IL2RG |
| Denosumab | Y | Y | N |  | N | 2010 | Giant cell tumor of the bone, Refractory Hypercalcemia of malignancy | TNFSF11, BRCA1 |
| Dexamethasone | Y | Y | N |  | Y | 1958 | Leukemia, Acute, Malignant Lymphomas, Multiple Myeloma (MM), Mycosis Fungoides (MF) | IGFBP1, CHKA, TDO2, THRSP, RPS19, CDH17, LOH19CR1, UBR1, SAG, TJP1, S100A10, CARTPT, SI, SDS, TEAD1, JUNB, PZP, SFTPA1, TSC22D3, DPEP1, IRS2, MT1F, ACTC1, TGFBR3, HYAL2, KRT19, FABP1, CRISPLD2 |
| Dexrazoxane | Y | Y | N |  | N | 1995 |  | TOP2A, MAPK8, CP, TOP2B, DPYS |
| Diethylstilbestrol | N | N | Y |  | N | 1973 |  | ESRRG, ESRRB, ESRRA, E2F1, NCOA2, PTGES3, LPO, AMH, NPPC, WNT7A |
| Dinutuximab | Y | Y | N |  | N | 2015 | High Risk Neuroblastoma | B4GALNT1, MYCN |
| Docetaxel | Y | Y | N |  | Y | 1996 | Esophageal Cancers, Ewing&#39;s Sarcoma, Locally Advanced Breast Cancer (LABC), Metastatic Bladder Cancer, Metastatic Breast Cancer, Metastatic Hormone Refractory Prostate Cancer, Metastatic Non-Small Cell Lung Cancer, Node Positive Breast Cancer, Ovarian Cancer Metastatic, Small Cell Lung Cancer (SCLC), Soft Tissue Sarcoma (STS), Advanced untreated gastric adenocarcinoma, Locally advanced Squamous cell carcinoma of head and neck, Locally advanced untreated non small cell lung cancer, Metastatic Squamous cell carcinoma of head and neck, Refractory, locally advanced Non small cell lung cancer, Refractory, metastatic Non small cell lung cancer | SLC10A2, EPHX1, CYP4B1, PGP, SOX10, BARD1, ATP7A, SPG7, XRCC4, TLE3, RNF8, ORM2, SULT1C4, MAP4, RPL13, CHST3, MUC16, SNORD68, MDM4, VAC14, ABCC6, MAP2, PRDX4, GAS6 |
| Doxorubicin | N | Y | Y |  | Y | 1974 | Acute Lymphoblastic Leukaemias (ALL), Acute Myeloblastic Leukemia, Advanced Endometrial Cancer, Advanced Soft Tissue Sarcoma, Bladder transitional cell carcinoma, Carcinoma, Bronchogenic, Gastric Carcinoma, Kaposi&#39;s Sarcoma AIDS Related, Lymphoma, Hodgkins, Malignant Lymphomas, Metastatic Breast Cancer, Multiple Myeloma (MM), Mycosis Fungoides (MF), Neuroblastomas, Ovarian Cancer Metastatic, Ovarian Carcinoma, Sarcoma, Bone, Sezary Syndrome, Soft Tissue Sarcoma (STS), Thyroid Carcinoma, Waldenstrom&#39;s Macroglobulinemia (WM), Wilms&#39; tumor, Advanced Thymoma, Advanced uterine sarcoma | ZEB1, NOLC1, MYB, NCF4, RPSA, LRP1B, CYBA, GADD45A, PPM1D, TAF1, RAC2, AKR1A1, SLC22A16, CBR3, SDHB, H2AFX, AKR7A2, CBR1, SP3, SETD4 |
| Doxorubicin Liposome | Y | Y | N |  | N | 1974 | Acute Lymphoblastic Leukaemias (ALL), Acute Myeloblastic Leukemia, Advanced Endometrial Cancer, Advanced Soft Tissue Sarcoma, Bladder transitional cell carcinoma, Carcinoma, Bronchogenic, Gastric Carcinoma, Kaposi&#39;s Sarcoma AIDS Related, Lymphoma, Hodgkins, Malignant Lymphomas, Metastatic Breast Cancer, Multiple Myeloma (MM), Mycosis Fungoides (MF), Neuroblastomas, Ovarian Cancer Metastatic, Ovarian Carcinoma, Sarcoma, Bone, Sezary Syndrome, Soft Tissue Sarcoma (STS), Thyroid Carcinoma, Waldenstrom&#39;s Macroglobulinemia (WM), Wilms&#39; tumor, Advanced Thymoma, Advanced uterine sarcoma | ZEB1, NOLC1, MYB, NCF4, RPSA, LRP1B, CYBA, GADD45A, PPM1D, TAF1, RAC2, AKR1A1, SLC22A16, CBR3, SDHB, H2AFX, AKR7A2, CBR1, SP3, SETD4 |
| Durvalumab | Y | Y | N |  | N | 2017 | Unresectable Stage III Non-small Cell Lung Cancer, Urothelial carcinoma ureter metastatic, Locally advanced Urothelial Carcinoma, Unresectable, locally advanced PD-L1 positive Lung Cancer Non-Small Cell Cancer (NSCLC) | CD274, MSH2, PBRM1, EGFR, MSH6, ALK |
| Duvelisib | N | Y | N |  | N | 2018 | Relapsed or Refractory Chronic Lymphocytic Leukemia (CLL), Relapsed or Refractory Follicular Lymphoma, Refractory, relapsed small lymphocytic lymphoma | PIK3CD, PIK3CG |
| Edotreotide | Y | N | N |  | N | 2019 | Neuroendocrine Tumors | SSTR2 |
| Elotuzumab | Y | Y | N |  | N | 2015 | Refractory Multiple Myeloma | SLAMF7 |
| Emapalumab | N | Y | N |  | N | 2018 |  | IFNG, SH2D1A, RAB27A, PRF1, UNC13D, XIAP, STX11, STXBP2 |
| Enasidenib | N | Y | N |  | N | 2017 | Refractory Acute Myelogenous Leukemia (AML), Relapsed Acute Myelogenous Leukemia (AML) | IDH2, FLT3 |
| Encorafenib | Y | Y | N |  | N | 2018 | Metastatic Melanoma, Unresectable Melanoma | ARAF, RAF1, BRAF, CCND1, MAP2K1 |
| Entrectinib | Y | Y | N |  | N | 2019 | Metastatic Solid Tumors, Non-Small Cell Lung Carcinoma (NSCLC), Tumors, Solid | NTRK3, NTRK2, ROS1, ETV6 |
| Enzalutamide | Y | Y | N |  | N | 2012 | Metastatic Castration Resistant Prostate Cancer | AR, CYP17A1, PTEN |
| Epirubicin | N | Y | Y |  | N | 1999 | Breast Cancer, Breast Cancer Stage II, Breast Cancer Stage III, Colorectal Cancers, Hormone Refractory Prostate Cancer, Non-Small Cell Lung Carcinoma (NSCLC), Ovarian Cancer, Papillary transitional cell carcinoma of bladder, Recurrent Superficial Bladder Cancer, Small Cell Lung Cancer (SCLC), Soft Tissue Sarcoma (STS), Stomach Tumor, Carcinoma in situ of urinary bladder | CHD1, IRS1, MDM4, PERP, GNL3, CBR3, TP53AIP1, CCNK, PPP2R5D, HMMR, FOXO1, RBX1, FOXP3 |
| Erdafitinib | N | Y | N |  | N | 2019 | Locally Advanced, Susceptible FGFR3 or FGFR2 genetic alterations, Condition has progressed during or following at least one line of prior platinum- containing chemotherapy including within 12 months of neoadjuvant or adjuvant platinum-containing chemotherapy urothelial carcinoma, Metastatic Susceptible FGFR3 or FGFR2 genetic alterations, Condition has progressed during or following at least one line of prior platinum- containing chemotherapy including within 12 months of neoadjuvant or adjuvant platinum-containing chemotherapy Metastatic Urothelial Carcinoma | FGFR2, FGFR1, FGFR4, FGFR3 |
| Eribulin | Y | Y | N |  | N | 2010 | Metastatic Liposarcoma, Refractory, metastatic Breast cancer, Unresectable Liposarcoma | TUB, TUBB1, BCL2 |
| Erlotinib | Y | Y | N |  | Y | 2004 | Locally Advanced Non-Small Cell Lung Cancer, Locally Advanced Pancreatic Cancer, Metastatic Non-Small Cell Lung Cancer, Non-Small Cell Lung Carcinoma (NSCLC), Pancreatic Cancer Metastatic | EGFR, CDH1, DDR2, CBL, ERRFI1 |
| Estramustine | N | Y | Y |  | N |  | Metastatic Hormone Refractory Prostate Cancer | MAP1A, MAP2, KLK3, HEXB |
| Etoposide | N | Y | Y |  | Y | 1983 | Acute Lymphoblastic Leukaemias (ALL), Acute Myeloid Leukemia (AML), Advanced Hodgkin&#39;s Lymphoma, Ewing&#39;s Sarcoma, Merkel cell cancer, Multiple Myeloma (MM), Neuroblastomas, Neuroendocrine Tumours, Non-Hodgkin&#39;s Lymphoma (NHL), Non-Small Cell Lung Carcinoma (NSCLC), Ovarian Cancer, Prostate Cancer, Retinoblastoma, Sarcoma, Osteogenic, Small Cell Lung Cancer (SCLC), Wilms&#39; tumor, Locally advanced Thymoma, Metastatic Thymic Cancer, Refractory Sarcoma, Refractory Testicular cancer | TOP2A, TOP2B, SLIT1, PAPOLA, EIF4E, TNFSF13B, MUC16, GDF15, BTF3P11, DYNC2H1, PLA2G6, MYCN, ACTR2, UGT1A10, AFP, E2F1, UGT1A1, PGP |
| Everolimus | Y | Y | N |  | N | 2009 | Subependymal giant cell astrocytoma, tuberous sclerosis complex, Advanced Carcinoid tumor, Locally advanced gastrointestinal origin Progressive Neuroendocrine Tumors, Locally advanced lung origin Progressive Neuroendocrine Tumors, Metastatic gastrointestinal origin Progressive Neuroendocrine Tumors, Metastatic lung origin Progressive Neuroendocrine Tumors, Pancreatic origin Progressive Neuroendocrine Tumors, Refractory Advanced Renal Cell Carcinoma, Refractory Waldenstrom&#39;s Macroglobulinaemia, Refractory, advanced Breast cancer, Unresectable gastrointestinal origin Progressive Neuroendocrine Tumors, Unresectable lung origin Progressive Neuroendocrine Tumors | MTOR, FKBP1A, GNAQ, RPTOR, STK11, NF1, NF2, BAP1, KDM5C, PTEN, FLCN, VHL, TSC2, TSC1, MPL, PBRM1, GNA11 |
| Exemestane | N | Y | Y |  | N | 1999 | Early Breast Cancer, Refractory, advanced Breast cancer | CYP19A1, AKR1C3, AKR1C4, KMT2C, CYP1B1, BGLAP |
| Fedratinib | N | Y | N |  | N | 2019 |  | JAK1, BRD4, JAK2, TYK2 |
| Fludarabine | N | Y | Y |  | Y | 1991 | B-cell chronic lymphocytic leukemia/prolymphocytic leukemia/small lymphocytic lymphoma refractory, Refractory Non-Hodgkin&#39;s lymphoma | RRM1, POLA1, DCK, IKZF1, SULT2B1, CXCL12, SLC22A12, SLC29A1, MPO, CD40, XIAP, CYP2E1 |
| Fluorouracil | N | Y | Y |  | Y | 1962 | Breast Cancer, Malignant Neoplasm of Colon, Malignant Neoplasm of Pancreas, Malignant Neoplasm of Stomach, Rectal Carcinoma, Superficial Basal Cell Carcinoma | ABCC11, GALNT14, KLC1, IL11, ENOSF1, TYMP, ALCAM, CBLB, IGFBP3, XRCC3, DLG5, EXO1, DPYS, DPYD, ABCG1, CYP2A6, RGS5, TPT1, PARD3B, NT5C1A, MYB, WNT5B, XRCC4, MUC2, MTHFR, MIR27A, CDC25C, LGR5, FPGS, UMPS, TMEM167A, CHN2, MEGF11 |
| Fluoxymesterone | N | Y | N |  | N | 1956 | Inoperable, metastatic Breast cancer | ESR1, NR3C1, HSD11B2, PRL |
| Flutamide | N | Y | Y |  | N | 1989 | C Prostatic Carcinoma, Stage B2 Prostatic Carcinoma, Stage D2 Prostatic carcinoma | TH, PEBP1, CDKN1A, IL2RA, AQP9, VIP, KLK3, MPO |
| Fotemustine | Y | N | N |  | N |  | Metastatic Melanoma | TXNRD1, MGMT |
| Fulvestrant | Y | Y | N |  | N | 2002 | Advanced or Metastatic Breast Cancer, Refractory, advanced Breast cancer, Refractory, metastatic Breast cancer | ESR1, GPER1, ESR2, TFPI, CDK6, CCNE1, EPHX2, ESRRA |
| Gefitinib | Y | Y | N |  | N | 2003 | Metastatic Non-Small Cell Lung Cancer | EGFR, NFKBIB, CRKL, CEACAM5, PBK, TAB2, EMP1 |
| Gemcitabine | N | Y | Y |  | Y | 1996 | Advanced Ovarian Cancer, Carcinoma Breast Stage IV, Locally Advanced Biliary Tract Cancer, Locally Advanced Non-Small Cell Lung Cancer, Locally Advanced Pancreatic Adenocarcinoma, Metastatic Biliary Tract Cancer, Metastatic Bladder Cancer, Non-small Cell Lung Cancer (NSCLC), Stage IV, Advanced Bladder cancer, Stage 4 Pancreatic adenocarcinoma | CMPK1, RRM1, CBL, SLC28A3, ATAT1, SLC28A1, CD3EAP, SLC29A2, SMAD4, SLC29A1, NT5C1A, SLC29A3, CDA, SERPINA5, PRRC2A, SH2D5, GPX5, NT5C3A, CDSN, ALG10, XYLT2, DCTD, MSH5, NT5C, PPP1R13L, CDC5L, ETS2, PPP1R18, CIP2A, DOCK8, SLC28A2, PAPD7, DCK, DPCR1, DAPK1, C6ORF15, POLR2A, PSORS1C1, FANCC, FHL2, NT5C2, HCP5 |
| Gemtuzumab Ozogamicin | Y | Y | N |  | N | 2000 | Acute Myeloid Leukemia (AML), Refractory Acute Myelogenous Leukemia (AML), Relapsed Acute Myelogenous Leukemia (AML) | ABCB1, CD33 |
| Gilteritinib | Y | Y | N |  | N | 2018 | Relapsed or Refractory Acute Myeloid Leukemia With FLT3 Activating Mutations | AXL, ROS1, LTK, FLT3, MERTK, ALK |
| Glasdegib | Y | Y | N |  | N | 2018 | Acute Myeloid Leukemia (AML) | SMO, MTOR |
| Goserelin | N | Y | Y |  | N | 1989 | Advanced Breast Cancer, Early Breast Cancer, Advanced carcinoma of the prostate, Stage M1 hormone dependent carcinoma of the prostate, Stage T2b carcinoma of the prostate, Stage T2c carcinoma of the prostate, Stage T3 carcinoma of the prostate, Stage T4 carcinoma of the prostate | LHCGR, GNRHR, MYOD1, PGR, GNRH1 |
| Histamine dihydrochloride | Y | Y | N |  | N | 1939 |  | HRH4, HRH2, CA5A, HRH3, CA7, SLC29A4, SLC18A2, LMNA |
| Histrelin | N | Y | N |  | N | 1991 | Advanced Prostate Cancer | GNRHR, GNRHR2 |
| Hydroxycarbamide | N | Y | N |  | Y | 1967 | Essential Thrombocythemia (ET), Head and Neck Carcinoma, Melanoma, Ovarian Cancer Metastatic, Polycythemia Vera (PV), Chronic, refractory Myeloid Leukemia, Inoperable Ovarian cancer | RRM2, RRM1, RRM2B, CP, DNTT, MAP3K5, DUSP1, EIF4E, PTH, DNMT1, HRAS |
| Ibritumomab Tiuxetan | Y | Y | N |  | N | 2002 | Follicular Non-Hodgkin&#39;s Lymphoma, Follicular Non-Hodgkin&#39;s Lymphoma Refractory | MS4A1 |
| Ibrutinib | Y | Y | N |  | N | 2013 | Chronic Lymphocytic Leukaemia (CLL), Mantle Cell Lymphoma (MCL), Waldenstrom&#39;s Macroglobulinemia (WM) | TEC, BTK, BMX, TXK, PLCG2, MYD88 |
| Idarubicin | N | Y | Y |  | N | 1990 | Acute Myeloid Leukemia (AML) | NT5C3A, ETV6, RAC2, SLC22A12, SULT2B1, NCF4, DNMT3A, DCK, CYBA |
| Idelalisib | Y | Y | N |  | N | 2014 | Relapsed Chronic Lymphocytic Leukemia, Relapsed Small Lymphocytic Lymphoma, Relapsed follicular B-cell non-Hodgkin lymphoma | PIK3CA, PIK3CD |
| Ifosfamide | N | Y | Y |  | Y | 1988 | Cancer, Bladder, Cervical Cancers, Ewing&#39;s Sarcoma, Head and Neck Carcinoma, Lymphoma, Hodgkins, Malignant Neoplasm of Pancreas, Non-Hodgkin&#39;s Lymphoma (NHL), Ovarian Cancer, Sarcoma, Osteogenic, Small Cell Lung Cancer (SCLC), Soft Tissue Sarcoma (STS), Testicular Germ Cell Cancer, Advanced thymic carcinoma | IL6, DNMT1, CYP2A6, GSTP1, HSPA4, MGMT, CYP2B6 |
| Imatinib | Y | Y | N |  | Y | 2001 | Chordomas, Chronic Eosinophilic Leukemia (CEL), Chronic Myeloid Leukemia (CML), Desmoid Tumors, FIP1L1-PDGFRα fusion kinase status unknown Chronic eosinophilic leukemia, Metastatic Gastrointestinal Stromal Tumor, Metastatic Melanoma, Myelodysplastic Syndromes (MDS), Refractory Acute Lymphoblastic Leukemia, Metastatic Dermatofibrosarcoma protuberans, Newly diagnosed Acute Lymphoblastic Leukaemia, Newly diagnosed, chronic phase Chronic myeloid leukemia, Recurrent Dermatofibrosarcoma protuberans, Refractory, accelerated phase Chronic myeloid leukemia, Refractory, blast crisis Chronic myeloid leukemia, Refractory, chronic phase Chronic myeloid leukemia, Systemic mastocytosis with associated hematological neoplasm, Unresectable Gastrointestinal stromal tumor | ABL1, PDGFRB, KIT, PDGFRA, BCR, DDR1, CSF1R, ABCB4, PDGFB, NF2, ULK3, CHST1, CYP2F1, SLC22A5, BCL2L11, SLC22A4, SLCO1A2, UGT2A1, RCSD1, FIP1L1, IKZF1, SFN, SLC22A1 |
| Imiquimod | Y | Y | N |  | N | 1997 | Superficial Basal Cell Carcinoma | TLR7, TLR8, IFNA1 |
| Inotuzumab Ozogamicin | Y | Y | N |  | N | 2017 | Refractory B-cell precursor Acute Lymphoblastic Anemia, Relapsed B-cell precursor Acute Lymphoblastic Anemia | CD22 |
| Interferon Alfa-2b | Y | Y | N |  | N | 1986 | Hairy Cell Leukemia (HCL), Kaposi&#39;s sarcoma, Melanoma, Malignant | IFNAR1, IFNAR2 |
| Iobenguane | N | Y | N |  | N | 1994 | Metastatic Pheochromocytoma, Metastatic Neuroblastoma, Primary Neuroblastomas, Primary Pheochromocytomas, Unresectable, locally advanced iobenguane-scan positive Paraganglioma, Unresectable, locally advanced iobenguane-scan positive Pheochromocytomas, Unresectable, metastatic iobenguane-scan positive Paraganglioma, Unresectable, metastatic iobenguane-scan positive Pheochromocytomas | TH, SLC6A2 |
| Ipilimumab | Y | Y | N |  | N | 2011 | Advanced Renal Cell Carcinoma, Cutaneous Melanoma, Hepatocellular Carcinoma, Metastatic Melanoma, Unresectable Melanoma, Metastatic MSI-H/dMMR colorectal cancer | CTLA4, PBRM1, MDM2, HLA-A, NRAS |
| Irinotecan | N | Y | Y |  | Y | 1996 | Esophageal Cancers, Ewing&#39;s Sarcoma, Glioblastomas, Malignant Neoplasm of Pancreas, Malignant Neoplasm of Stomach, Metastatic Colorectal Carcinoma, Non-Small Cell Lung Carcinoma (NSCLC), Ovarian Cancer, Rhabdomyosarcomas, Small Cell Lung Cancer (SCLC), Recurrent, IV-B Cervical cancer, Recurrent, metastatic Colorectal carcinoma, Refractory, metastatic Pancreatic adenocarcinoma | TOP1, TOP1MT, ISG15, ABCC5, UGT1A9, XRCC3, C8ORF34, CES2, TGFBR2, ABCG1, RAD50, RUNX3, ATRX, AREG, SEMA3C, KLC1, SHMT1, UGT1A, UBE2I, KCNQ5, DCBLD1, RGS5, DKK1 |
| Irinotecan Liposome | Y | Y | N |  | N | 1996 | Esophageal Cancers, Ewing&#39;s Sarcoma, Glioblastomas, Malignant Neoplasm of Pancreas, Malignant Neoplasm of Stomach, Metastatic Colorectal Carcinoma, Non-Small Cell Lung Carcinoma (NSCLC), Ovarian Cancer, Rhabdomyosarcomas, Small Cell Lung Cancer (SCLC), Recurrent, IV-B Cervical cancer, Recurrent, metastatic Colorectal carcinoma, Refractory, metastatic Pancreatic adenocarcinoma | TOP1, TOP1MT, ISG15, ABCC5, UGT1A9, XRCC3, C8ORF34, CES2, TGFBR2, ABCG1, RAD50, RUNX3, ATRX, AREG, SEMA3C, KLC1, SHMT1, UGT1A, UBE2I, KCNQ5, DCBLD1, RGS5, DKK1 |
| Isatuximab | Y | Y | N |  | N | 2020 | Multiple Myeloma (MM) | CD38 |
| Ivosidenib | N | Y | N |  | N | 2018 | Refractory, relapsed Acute Myeloid Leukemia | IDH1 |
| Ixabepilone | N | Y | N |  | N | 2007 | Locally Advanced Breast Cancer (LABC), Metastatic Breast Cancer | TUBB3 |
| Ixazomib | Y | Y | N |  | N | 2015 | Multiple Myeloma (MM) | PSMC5, PSMB5, NPM1 |
| Lanreotide | N | Y | Y |  | N | 2007 | Unresectable, locally advanced enteropancreatic neuroendocrine tumors, Unresectable, locally advanced gastroenteropancreatic neuroendocrine tumors, Unresectable, metastatic enteropancreatic neuroendocrine tumors, Unresectable, metastatic gastroenteropancreatic neuroendocrine tumors | SSTR5, SSTR3, SSTR2, SSTR1, EDN1 |
| Lapatinib | Y | Y | N |  | N | 2007 | Metastatic Breast Cancer, Refractory, advanced Breast cancer, Refractory, metastatic Breast cancer | EGFR, ERBB2, ERBB4, ABCB11, CDH1, EEF2K, HLA-DRB1, NF2, NRG1, CDKN1B, ERBB3, HLA-DQA1 |
| Larotrectinib | Y | Y | N |  | N | 2018 | NTRK1 Fusion Positive, NTRK2 Fusion Positive, NTRK3 Fusion Positive | NTRK3, NTRK2, NTRK1, ETV6 |
| Lenalidomide | Y | Y | N |  | Y | 2005 | Chronic Lymphocytic Leukemia (CLL) - Refractory, Mantle Cell Lymphoma (MCL), Multiple Myeloma (MM), Myelodysplastic Syndrome, Refractory Diffuse Large B Cell Lymphoma | CUL4A, TNFSF11, CRBN, CDH5, DDB1, RBX1, CTNNB1, IKZF1, FGF2, VEGFB, VEGFC |
| Lenvatinib | Y | Y | N |  | N | 2015 | Advanced Renal Cell Carcinoma, Locally recurrent radioactive iodine-refractory Thyroid cancer, Metastatic radioactive iodine-refractory Thyroid cancer, Progressive radioactive iodine-refractory Thyroid cancer | FLT4, FGFR2, RET, FGFR4, FGFR1 |
| Letrozole | N | Y | Y |  | N | 1997 | Advanced Breast Cancer, Early Breast Cancer, Ovarian Epithelial Cancer, Advanced HR + HER2 - breast cancer, Metastatic HR + HER2 - breast cancer | CYP19A1, MMP2, CDKN2A, CYP2A6, CYP1B1, TNFRSF11B, KMT2C, TNFSF11 |
| Leuprolide | N | Y | N |  | N | 1985 | Advanced Prostate Cancer | GNRHR, GNRH1 |
| Lomustine | N | Y | Y |  | N | 1976 | Primary Brain Tumors, Refractory Hodgkin Lymphoma, Tumors Metastatic to Brain | STMN4, MGMT, ADK, ATRX |
| Lorlatinib | Y | Y | N |  | N | 2018 | Progressive, metastatic Anaplastic lymphoma kinase-positive Metastatic Non-Small Cell Lung Cancer | ROS1, FES, ALK, EML4, NPM1, RB1 |
| Lu 177-Dotatate | Y | Y | N |  | N | 2018 | Gastroenteropancreatic Neuroendocrine Tumors | SSTR5, SSTR2, SSTR4, SSTR1, SSTR3 |
| Lurbinectedin | N | Y | N |  | N | 2020 | Metastatic Small Cell Lung Cancer |  |
| Medroxyprogesterone | N | Y | Y |  | N | 1959 | Metastatic Renal Cell Carcinoma, Metastatic Endometrial carcinoma | PGR, MMP1, CYP1B1 |
| Megestrol | N | Y | Y |  | N | 1971 | Advanced Breast Cancer, Advanced Endometrial Cancer | PGR |
| Melphalan | N | Y | Y |  | Y | 1964 | Multiple Myeloma (MM), Ovarian Epithelial Cancer, Severe Hodgkin Lymphoma | MGMT, PLAT, SLC7A5, CDKN1A, OPLAH, FANCC, IFNG, CTLA4 |
| Mercaptopurine | Y | Y | N |  | Y | 1953 | Acute Lymphoblastic Leukaemias (ALL), Acute Promyelocytic Leukemia (APL), Lymphoma, Lymphoblastic | IMPDH1, HPRT1, PPAT, ITPA, GATA3, PACSIN2, GNMT, HLA-DQA1, NUDT15, PRPS1, FTO, DROSHA, FOLH1, NT5C2, TPMT, SLC29A2, VCAM1, MTHFR |
| Methotrexate | Y | Y | N |  | Y | 1953 | Acute Lymphocytic Leukemia (ALL), Acute Promyelocytic Leukemia (APL), Breast Cancer, Cancer, Bladder, Central Nervous System Lymphoma, Choriocarcinoma, Head and Neck Carcinoma, Meningeal leukemia, Sarcoma, Osteogenic, Small Cell Lung Cancer (SCLC), Soft Tissue Sarcoma (STS), Squamous Cell Carcinoma of Lung, Advanced Alibert-Bazin syndrome, Advanced non-Hodgkin lymphoma, Nonleukemic meningeal cancer | DHFR, ATIC, IL1RN, MTRR, ATP5E, FOXP3, NALCN, S100A8, SLC46A1, SPECC1L, HLA-G, FPGS, PACSIN2, KIR2DS4, SLC16A7, ITPA, ADORA2A-AS1, SLC22A9, SHMT1, ENOSF1, ARID5B, GGH, KLRD1, TNFAIP3, PTPRM, SLAMF1, AMPD1, ICAM3 |
| Midostaurin | Y | Y | N |  | N | 2017 | Acute Myeloid Leukemia (AML), Malignant mast cell neoplasm, Systemic mastocytosis with associated hematological neoplasm | KIT, PRKCA, PRKCH, PRKD1, FLT3, ETV6, NPM1, STK32B |
| Mifamurtide | Y | N | N |  | N | 2009 | High-grade, nonmetastatic Osteosarcoma, Resectable, nonmetastatic Osteosarcoma | TLR4, NOD2 |
| Mitomycin | N | Y | Y |  | N | 1981 | Adenocarcinoma of the Pancreas, Adenocarcinoma of the Stomach, Breast Cancer, Cancer, Anal, Cancer, Bladder, Cervical Cancers, Head and Neck Carcinoma, Mesothelioma, Non-Small Cell Lung Carcinoma (NSCLC), Low-grade Upper Tract Urothelial Cancer (LG-UTUC) | BGLAP, PALB2, FANCC, VWF |
| Mitoxantrone | N | Y | Y |  | N | 1987 | Acute Lymphocytic Leukemia (ALL), Acute Myeloid Leukemia (AML), Acute Promyelocytic Leukemia (APL), Lymphoma, Hodgkins, Metastatic Breast Cancer, Non-Hodgkin&#39;s Lymphoma (NHL), Relapsed Leukemia, Relapsed Lymphomas, Hormone refractory, advanced Prostate cancer, Relapsed Hepatocellular carcinoma | UGT1A5, C10ORF67, GALNT14, RPSA |
| Mogamulizumab | Y | Y | N |  | N | 2018 | Refractory Mycosis Fungoides/Sezary Syndrome, Relapsed Mycosis Fungoides/Sezary Syndrome | CCR4 |
| Moxetumomab Pasudotox | Y | Y | N |  | N | 2018 | Relapsed/Refractory Hairy Cell Leukemia | CD22, EEF2 |
| Nab-Paclitaxel | Y | Y | N |  | N | 1992 | Advanced Cervical Cancer, Advanced Head and Neck Cancer, Advanced Ovarian Cancer, Advanced Soft Tissue Sarcoma, Esophageal Cancers, Fallopian Tube Cancer, Kaposi&#39;s sarcoma, Locally Advanced Non-Small Cell Lung Cancer, Malignant Neoplasm of Stomach, Malignant Peritoneal Neoplasm, Metastatic Bladder Cancer, Metastatic Breast Cancer, Metastatic Melanoma, Metastatic Non-Small Cell Lung Cancer, Non-Small Cell Lung Carcinoma (NSCLC), Ovarian Cancer, Pancreatic Adenocarcinoma Metastatic, Advanced Bladder cancer, Advanced Thymoma, Metastatic Penile cancer, Refractory Small cell lung cancer, Refractory Testicular germ cell cancer | TUBB1, STMN1, FGD4, PDCD4, DSCAM, EPHA5, MAP4, EIF4EBP1, MYB, TUBB4A, TUBA3C, TLE3, SPATA5, NFE2, MAD1L1, SOX10, MAP2, TRIM5, ABCC10 |
| Necitumumab | Y | Y | N |  | N | 2015 | Metastatic Non-Small Cell Lung Cancer | EGFR, KRAS |
| Nelarabine | Y | Y | N |  | N | 2005 | Lymphoblastic lymphoma (Precursor T-lymphoblastic lymphoma/leukemia) refractory, Refractory Acute Lymphoblastic Leukemia | POLA1 |
| Neratinib | Y | Y | N |  | N | 2017 | Breast Cancer | EGFR, ERBB2, HERC2 |
| Nilotinib | Y | Y | N |  | Y | 2007 | Chronic Phase Chronic Myeloid Leukemia, Refractory Gastrointestinal stromal tumor, Refractory, accelerated phase Chronic myeloid leukemia | ABL1, DDR1, KIT, DDR2, NF1, BCR, UGT1A1 |
| Nilutamide | N | Y | N |  | N | 1996 | Metastatic Hormone Refractory Prostate Cancer | KLK3 |
| Nintedanib | Y | Y | N |  | N | 2014 | Locally advanced Non-Small Cell Lung Cancer (NSCLC) caused by Adenocarcinoma, Locally recurrent Non-Small Cell Lung Cancer (NSCLC) caused by Adenocarcinoma, Metastatic Non-Small Cell Lung Cancer (NSCLC) caused by Adenocarcinoma | LYN, FLT4, FGFR2, FGR, PDGFRA, FLT1, PDGFRB, FGFR3, FGFR1, CFLAR |
| Niraparib | Y | Y | N |  | N | 2017 | Fallopian Tube Cancer, Ovarian Epithelial Cancer, Primary Peritoneal Cancer | PARP1, PARP2, BRCA1, SLFN11, BRCA2, ATR |
| Nivolumab | Y | Y | N |  | Y | 2015 | Advanced Renal Cell Cancer, Classical Hodgkin&#39;s Lymphoma, Hepatocellular Carcinoma, Low Risk Advanced Renal Cell Cancer, Melanoma, Metastatic Colorectal Cancer (MCRC), Metastatic Melanoma, Metastatic Non-Small Cell Lung Cancer, Metastatic Small Cell Lung Cancer, Metastatic Squamous Cell Carcinoma of the Head and Neck, Metastatic Urothelial Carcinoma (UC), Recurrent Head and Neck Squamous Cell Carcinoma, Unresectable Melanoma, Intermediate risk Advanced Renal Cell Cancer, Locally advanced Urothelial Carcinoma | PDCD1, STK11, ARID1A, MDM2, HLA-DRA, MDM4, PMS2, CDK12, PBRM1, ALK, CD274 |
| Obinutuzumab | Y | Y | N |  | N | 2013 | Refractory Follicular Lymphoma, Previously untreated Chronic lymphocytic leukemia | MS4A1 |
| Ofatumumab | Y | Y | N |  | N | 2009 | Chronic Lymphocytic Leukaemia (CLL), Chronic Lymphocytic Leukemia (CLL) - Refractory, Progressive Chronic Lymphocytic Leukaemia (CLL), Recurrent Chronic Lymphocytic Leukaemia (CLL) | MS4A1 |
| Olaparib | Y | Y | N |  | N | 2014 | Fallopian Tube Cancer, Malignant Peritoneal Neoplasm, Metastatic Breast Cancer, Ovarian Epithelial Cancer, Primary Peritoneal Cancer, Advanced deleterious germline or somatic BRCA-mutated advanced epithelial ovarian cancer, Advanced deleterious germline or somatic BRCA-mutated fallopian tube cancer, Advanced deleterious germline or somatic BRCA-mutated peritoneal cancer, Refractory Advanced Ovarian Cancer | PARP2, PARP3, PARP1, IDH2, ATR, BARD1, CHEK2, FANCA, CBLC, BRCA2, BAP1, VHL, STAG2, BRCA1, CDK12, PALB2, ATM |
| Olaratumab | Y | Y | N |  | N | 2016 | Soft Tissue Sarcoma (STS) | PDGFRA |
| Omacetaxine Mepesuccinate | N | Y | N |  | N | 2009 | Refractory, accelerated phase Chronic myeloid leukemia, Refractory, chronic phase Chronic myeloid leukemia | TERT, BCR, BIRC5 |
| Osimertinib | Y | Y | N |  | N | 2015 | Metastatic Non-Small Cell Lung Cancer | EGFR, ERBB2, MET, KRAS, NRAS |
| Oxaliplatin | N | Y | Y |  | Y | 2002 | Advanced Colorectal Cancer, Advanced Ovarian Cancer, Advanced Pancreatic Cancer, Chronic Lymphocytic Leukemia (CLL) - Refractory, Colon Cancer Stage III, Esophageal Cancers, Malignant Neoplasm of Stomach, Advanced biliary adenocarcinoma, Refractory Neuroendocrine Tumour, Refractory Non-Hodgkin&#39;s lymphoma, Refractory Testicular cancer | DPYD, GSTP1, ATP7B, ZEB1, CSMD1, DKK1, SELE, ABCC5, ATP7A, KLC3, MT1A, CXCL10, MTHFR, MGAT4A, FOXC2, ERCC1, PARD3B, GNA13, ERCC2 |
| Paclitaxel | Y | Y | N |  | Y | 1992 | Advanced Cervical Cancer, Advanced Head and Neck Cancer, Advanced Ovarian Cancer, Advanced Soft Tissue Sarcoma, Esophageal Cancers, Fallopian Tube Cancer, Kaposi&#39;s sarcoma, Locally Advanced Non-Small Cell Lung Cancer, Malignant Neoplasm of Stomach, Malignant Peritoneal Neoplasm, Metastatic Bladder Cancer, Metastatic Breast Cancer, Metastatic Melanoma, Metastatic Non-Small Cell Lung Cancer, Non-Small Cell Lung Carcinoma (NSCLC), Ovarian Cancer, Pancreatic Adenocarcinoma Metastatic, Advanced Bladder cancer, Advanced Thymoma, Metastatic Penile cancer, Refractory Small cell lung cancer, Refractory Testicular germ cell cancer | TUBB1, STMN1, FGD4, PDCD4, DSCAM, EPHA5, MAP4, EIF4EBP1, MYB, TUBB4A, TUBA3C, TLE3, SPATA5, NFE2, MAD1L1, SOX10, MAP2, TRIM5, ABCC10 |
| Padeliporfin | Y | N | N |  | N | 2017 | Low-Risk Prostate Cancer of the one lobe only |  |
| Palbociclib | Y | Y | N |  | N | 2015 | Advanced Breast Cancer, Metastatic Breast Cancer, Refractory, advanced Breast cancer, Refractory, metastatic Breast cancer | CDK6, CDK4, CDKN2A, SMARCA4, SMARCB1, CDKN2B, RB1, CCND2, CCNE1, CCND3 |
| Panitumumab | Y | Y | N |  | N | 2006 | Metastatic Colorectal Cancer (MCRC) | EGFR, NRG3, NRG2, HBEGF, TGFA, BTC, BRAF, EGF, KRAS, AREG, NRAS, MAP2K1, EREG, CDKN2A |
| Panobinostat | Y | Y | N |  | N | 2015 | Refractory Multiple Myeloma | HDAC8, HDAC7, HDAC1, HDAC6, HDAC2, HDAC9, HDAC4, HDAC3, HDAC11, MYCN, SMARCB1, BAP1 |
| Pazopanib | Y | Y | N |  | N | 2009 | Advanced Renal Cell Carcinoma, Advanced Soft Tissue Sarcoma, Advanced Thyroid cancer | FGF1, SH2B3 |
| Pegaspargase | Y | Y | N |  | Y | 1994 | Acute Lymphoblastic Leukaemias (ALL) | LINC00251, GATA3, BMP7, DOK5 |
| Peginterferon Alfa-2b | Y | Y | N |  | N | 2001 | Melanoma | IFNAR2, IFNAR1 |
| Pembrolizumab | Y | Y | N |  | N | 2014 | Advanced Renal Cell Carcinoma, Cervical Cancers, Colorectal Cancers, Metastatic Melanoma, Metastatic Solid Tumors, Unresectable Melanoma, Urothelial carcinoma ureter metastatic, Locally advanced Urothelial Carcinoma, Locally advanced gastroesphageal juntion adenocarcinoma, Metastatic gastroesphageal juntion adenocarcinoma, Metastatic nonsquamous non-small cell lung cancer, Recurrent, metastatic Head and Neck Squamous Cell Carcinoma, Refractory, metastatic Non small cell lung cancer, Refractory, relapsed Hodgkin Lymphoma, Refractory, relapsed Mediastinal Large B-cell Lymphoma, Unresectable Solid Tumors | PDCD1, PDCD1LG2, B2M, MDM2, CDK12, PBRM1, STK11, ARID1A, CD274, MDM4, POLE, HLA-DRA, JAK1 |
| Pemetrexed | Y | Y | N |  | N | 2004 | Mesothelioma, Ovarian Cancer, Pleural Mesotheliomas, Urothelial carcinoma ureter metastatic, Locally advanced nonsquamous non-small cell lung cancer, Metastatic nonsquamous non-small cell lung cancer, Recurrent, IV-B Cervical cancer, Unresectable Thymoma | DHFR, GART, TYMS, ATIC, FOLR3, SLC46A1, MTHFR, GGH, SLC19A1 |
| Pemigatinib | N | Y | N |  | N | 2020 | Unresectable, locally advanced Cholangiocarcinomas, Unresectable, metastatic Cholangiocarcinomas | FGFR2, FGFR1, FGFR3, FGFR4 |
| Pentostatin | N | N | Y |  | N | 1991 | B-Lymphocytic, prolymphocytic leukemia (Kiel Classification) refractory, Chronic Lymphocytic Leukaemia (CLL), Hairy Cell Leukemia (HCL), Mycosis Fungoides (MF), Sezary Syndrome | ADA, PRKAA1 |
| Pertuzumab | Y | Y | N |  | N | 2012 | Early Breast Cancer, Inflammatory Breast Cancer (IBC), Locally Advanced Breast Cancer (LABC), Metastatic Breast Cancer | ERBB2, PGR, NRG1, ERBB3 |
| Pertuzumab, Trastuzumab, Hyaluronidase | Y | Y | N |  | N |  |  |  |
| Pipobroman | N | N | Y |  | N | 1966 |  |  |
| Pirarubicin | N | N | N | Japan | N |  |  | ABCC1 |
| Pixantrone | Y | N | N |  | N | 2012 | Non-Hodgkin&#39;s Lymphoma, Relapsed, Refractory Non-Hodgkin&#39;s lymphoma | TOP2B |
| Plicamycin | N | Y | N |  | N | 1970 |  | BIRC5 |
| Polatuzumab Vedotin | Y | Y | N |  | N | 2019 | Diffuse Large B-Cell Lymphoma (DLBCL) | CD79B |
| Polyestradiol Phosphate | N | N | Y |  | N | 1957 |  | ESR1 |
| Pomalidomide | Y | Y | N |  | N | 2013 | Refractory Multiple Myeloma | CRBN, TNF, CUL4A, DDB1, RBX1 |
| Ponatinib | Y | Y | N |  | N | 2012 | Accelerated phase chronic myologenic leukemia, Acute Lymphoblastic Leukaemias (ALL), Chronic Phase Chronic Myeloid Leukemia, Blast phase Chronic myelocytic leukemia | RIPK2, FGFR4, RET, FLT3, FGFR3, FGFR1, RIPK1, FGFR2, PDGFRA, BCR, RIPK3, ABL1, KIT, ABI1, ABL2 |
| Porfimer | Y | Y | N |  | N | 1995 | Esophageal Cancers, Completely obstructive Non-small cell lung cancer, Microinvasive endobrachial Non-small cell lung cancer, Partially obstructive Non-small cell lung cancer |  |
| Pralatrexate | Y | Y | N |  | N | 2009 | Cutaneous T-Cell Lymphoma (CTCL), Relapsed Peripheral T-Cell Lymphoma, Refractory Peripheral T-cell Lymphoma Unspecified | DHFR, TYMS, PDF, RFC1 |
| Pralsetinib | N | Y | N |  | N | 2020 |  | RET |
| Prednisolone | Y | Y | N |  | Y | 1955 | Hypercalcemia of Malignancy, Leukemia, Acute, Malignant Lymphomas, Mycosis Fungoides (MF) | TAT, PNPLA3, NOTCH1, TG, CD34, ALPP, LOH19CR1, LCAT, DSE, TSPYL1, HMGB1 |
| Prednisone | N | Y | Y |  | N | 1955 | Acute Lymphoblastic Leukaemias (ALL), Aggressive Lymphoma, Hypercalcemia of Malignancy, Leukemia, Acute, Leukemias, Malignant Lymphomas, Metastatic Castration Resistant Prostate Cancer, Mycosis Fungoides (MF) | TPMT, PTPRC, VWF, TG, DROSHA, CTLA4, LINC00251, BMP7, MC2R, DOK5, GATA3, GSTA1, MBP, CXCL12 |
| Procarbazine | N | Y | Y |  | Y | 1969 | Non-Hodgkin&#39;s Lymphoma (NHL), Oligodendrogliomas, Primary Central Nervous System Lymphoma (PCNSL), Stage III Hodgkin&#39;s Disease, Stage IV Hodgkin&#39;s Disease | MAOA, BAX, CSF2, ATRX |
| Racotumomab | N | N | N | Cuba, Argentina | N |  |  |  |
| Radium 223 Dichloride | Y | Y | N |  | N | 2013 |  |  |
| Raltitrexed | N | N | Y | Canada | N | 1996 | Advanced Colorectal Cancer, Pleural Mesotheliomas | FPGS, TYMS, ALB, CDKN1B, DHFR, BCL2, SLC19A1 |
| Ramucirumab | Y | Y | N |  | N | 2014 | Advanced Gastric Cancer, Advanced gastro-esophageal junction adenocarcinoma, Refractory, metastatic Colorectal cancer, Refractory, metastatic Non small cell lung cancer | KDR, PDGFRA, KRAS |
| Ranimustine | N | N | N | Japan | N |  |  |  |
| Regorafenib | Y | Y | N |  | N | 2012 | Hepatocellular Carcinoma, Metastatic Colorectal Cancer (MCRC), Metastatic Gastrointestinal Stromal Tumor, Locally advanced Gastrointestinal stromal tumor, Unresectable Gastrointestinal stromal tumor | RAF1, DDR2, KIT, RET, TEK, FRK, MAPK11, EPHA2, ARAF, VHL, FBXW7, APC |
| Ribociclib | Y | Y | N |  | N | 2017 | Advanced Breast Cancer, Metastatic Breast Cancer | CDK6, CDK4, CCND2, NRAS, RB1, CCND1, CDKN2A, SMARCA4, CCND3, NOTCH1 |
| Ripretinib | N | Y | N |  | N | 2020 | Advanced Gastrointestinal Stromal Tumor (GIST) | KIT, PDGFRB, TEK, PDGFRA |
| Rituximab | Y | Y | N |  | Y | 1997 | Chronic Lymphocytic Leukaemia (CLL), Non-Hodgkin&#39;s Lymphoma (NHL) | MS4A1, FCGR2A, IL2, TGFB1, IL10, IL6, GSTA1, CXCL13, FCGR3A, LOH19CR1, XIAP, CXCL12 |
| Rituximab, Hyaluronidase | N | Y | N |  | N | 1997 | Chronic Lymphocytic Leukaemia (CLL), Non-Hodgkin&#39;s Lymphoma (NHL) | MS4A1, FCGR2A, IL2, TGFB1, IL10, IL6, GSTA1, CXCL13, FCGR3A, LOH19CR1, XIAP, CXCL12 |
| Romidepsin | Y | Y | N |  | N | 2009 | Refractory peripheral cutaneous T-cell lymphoma | HDAC1, HDAC3, HDAC4, HDAC9, HDAC2, HDAC6, HDAC8, HDAC5, HDAC7, CDKN2D, CDKN1A, CTAG1B |
| Rucaparib | Y | Y | N |  | N | 2016 | Advanced Ovarian Cancer | PARP3, PARP1, PARP2, PALB2, STAG2, SLC47A1, BRCA2, SLC47A2, BRCA1 |
| Ruxolitinib | Y | Y | N |  | N | 2011 | Post Polycythemia Vera Myelofibrosis, Refractory Polycythemia vera | JAK2, JAK3, JAK1, VHL, IL7R, MPL, CRLF2, CSF3R, PLAUR, SH2B3 |
| Sacituzumab Govitecan | N | Y | N |  | N | 2020 | Metastatic Triple-negative Breast Cancer |  |
| Selinexor | N | Y | N |  | N | 2019 | Refractory Multiple Myeloma | XPO1 |
| Selpercatinib | Y | Y | N |  | N | 2020 | Advanced RET-fusion thyroid cancer, Advanced RET-mutant medullary thyroid cancer, Metastatic RET-fusion Non Small Cell Lung Cancer, Metastatic RET-fusion thyroid cancer, Metastatic RET-mutant medullary thyroid cancer | RET, FLT1, FLT4, FGFR1, FGFR2, FGFR3 |
| Sipuleucel-T | N | Y | N |  | N | 2010 | Asymptomatic, metastatic hormone-refractory Prostate cancer, Minimally symptomatic, metastatic hormone-refractory Prostate cancer | ACPP |
| Sonidegib | Y | Y | N |  | N | 2015 | Refractory, locally advanced Basal cell carcinoma | SMO, MYCN, SUFU, PTCH1 |
| Sorafenib | Y | Y | N |  | N | 2005 | Advanced Renal Cell Carcinoma, Gastrointestinal Stromal Tumors, Hemangiosarcoma, Unresectable Hepatocellular Carcinoma, Locally recurrent refractory to radioactive iodine treatment Thyroid carcinoma, Metastatic refractory to radioactive iodine treatment Thyroid carcinoma | FLT3, BRAF, RAF1, KIT, ARAF, MAPK6, FGF3, PTPN6, GALNT14, MAPK4, MAPK7, ARID1A |
| Streptozocin | N | Y | N |  | N | 2005 | Stage 4 islet cell carcinoma | SLC2A2, SI, UCP1, SST, SULT1A2, ADCYAP1, SLC2A5, IGFBP1, VWF, ATF2, S100B, CDKN1B, GALP, SLC16A3, MT1H, THRSP, ACP5, CD40, SFTPB, HP, ENO2, RAMP3, LCAT, PREP, CYP7B1, CTSD, MGEA5, MYO9B, KCNA2 |
| Sunitinib | Y | Y | N |  | N | 2006 | Advanced Renal Cell Carcinoma, Soft Tissue Sarcoma (STS), Thyroid Cancers, Metastatic Pancreatic Neuroendocrine Tumors, Refractory Gastrointestinal stromal tumor, Unresectable, locally advanced Pancreatic Neuroendocrine Tumors | PDGFB, PDGFC, PDGFRB, FLT4, FLT3, PDGFD, KIT, KDR, FLT1, PDGFA, RET, PDGFRA, PTPRB, HMOX1, VHL, PBRM1, VEGFC, EWSR1, MKI67, BAP1, POR, PTPN12, IL13, KDM5C |
| Tafasitamab | N | Y | N |  | N | 2020 | Refractory Diffuse large B-cell lymphoma NOS, Relapsed Diffuse large B-cell lymphoma NOS | TNFRSF8 |
| Tagraxofusp | N | Y | N |  | N | 2018 | Blastic Plasmacytoid Dendritic Cell Neoplasm (BPDCN) | IL3RA, ARL2, CSF2RB |
| Talazoparib | Y | Y | N |  | N | 2018 | Locally Advanced Breast Cancer (LABC), Metastatic Breast Cancer | PARP2, PARP1, SLFN11, BRCA1, PSMD4, BRCA2 |
| Talimogene Laherparepvec | Y | Y | N |  | N | 2015 | Unresectable Skin Lesion |  |
| Tamoxifen | N | Y | Y |  | Y | 1977 | Breast Cancer, Desmoid Tumors, Endometrial Cancer, Invasive Breast Cancer, Invasive Breast Carcinoma, Metastatic Breast Cancer, Ovarian Cancer | EBP, FMO1, PLD1, E2F7, CCND1, PLD2, PIP, LRMDA, F5, MUC16, COL18A1, TFF3, ZNF423, RRAS2, FMO3 |
| Tazemetostat | N | Y | N |  | N | 2020 | Locally Advanced Epitheliod Sarcoma, Metastatic Epithelioid Sarcoma | EZH2, EZH1, SMARCB1, SMARCA4, KDM6A |
| Tegafur, Gimeracil, Oteracil | Y | N | N |  | N | 2011 | Advanced Gastric Cancer | TYMS, UMPS, DPYD |
| Tegafur, Uracil | N | N | N | Japan | N |  | Advanced Gastric Cancer | TYMS, UMPS, DPYD |
| Temoporfin | Y | N | N |  | N | 2001 | Advanced Head and Neck Squamous Cell Carcinoma |  |
| Temozolomide | Y | Y | N |  | N | 1999 | Advanced Melanoma, Glioblastoma Multiforme (GBM), Primary Central Nervous System Lymphoma (PCNSL), Refractory Ewing Sarcoma, Refractory Neuroblastoma, Soft Tissue Sarcoma (STS), Advanced Neuroendocrine tumor, Refractory Anaplastic astrocytoma, Refractory, advanced Mycosis fungoides, Refractory, advanced Sezary Syndrome | SLFN11, CSF2, STAG2, ATRX, ATR, H2AFX, MGMT, NOTCH1, BRCA2, MSH6 |
| Temsirolimus | Y | Y | N |  | N | 2007 | Advanced Renal Cell Carcinoma | MTOR, FKBP1A, CTNNB1, NF2, PIK3CA, PTEN, VHL, FBXW7 |
| Thalidomide | Y | Y | N |  | Y | 1998 | Multiple Myeloma (MM), Waldenstrom&#39;s Macroglobulinemia (WM), Treatment naive multiple myeloma | RBX1, ORM1, CRBN, CUL4A, DDB1, TNF, SPG7, XRCC5, GSTT1, CYP4B1, CHST3, ATP7A, ABCC6, VWF, RPL13, SULT1C4, IL6R, SLC10A2, SNORD68 |
| Thiotepa | Y | Y | N |  | N | 1959 | Adenocarcinoma of the Ovaries, Breast Adenocarcinoma, Papillary transitional cell carcinoma of bladder, Malignant effusion | CYP2B6 |
| Tioguanine | N | Y | N |  | Y | 1966 | Acute Nonlymphocytic Leukemia, Nonlymphocytic Acute myeloid leukemia | IMPDH2, IMPDH1, DOK5, GGH, TPM3, NUDT15, PRPS1, LINC00251, GDA, TPMT, NT5C2, GATA3, BMP7 |
| Tisagenlecleucel | Y | Y | N |  | N | 2017 | Refractory B-cell precursor acute lymphoblastic leukemia, Second or later relapsed B-cell precursor acute lymphoblastic leukemia | CD19, MS4A1 |
| Tivozanib | Y | N | N |  | N | 2017 | Advanced Renal Cell Carcinoma | FLT1, FLT4, KDR |
| Topotecan | Y | Y | N |  | N | 1996 | Acute Myeloid Leukemia (AML), Ewing&#39;s Sarcoma, Refractory Neuroblastoma, Metastatic Rhabdomyosarcoma, Recurrent, IV-B Cervical cancer, Refractory CNS lymphoma, Refractory CNS malignancy, Refractory, metastatic Ovarian cancer, Relapsed Small cell lung cancer | TOP1MT, TOP1, ATRX, RB1, MUC16, DDO |
| Toremifene | Y | Y | N |  | N | 1997 | Desmoid Tumors, Metastatic Breast Cancer | ESR1, PGR, CYP1B1, SHBG, TGFB1, CD80 |
| Trabectedin | Y | Y | N |  | N | 2015 | Metastatic Leiomyosarcoma, Metastatic Liposarcoma, Relapsed platinum-sensitive Ovarian cancer, Unresectable Leiomyosarcoma, Unresectable Liposarcoma | FGFR2, PARP1 |
| Trametinib | Y | Y | N |  | N | 2013 | Metastatic Melanoma, Unresectable Melanoma | MAP2K1, MAP2K5, MAP2K2, MAP2K7, GNA11, ARAF, ATXN1L, ETV5, ETV4, ETV1, NF1, NRAS, DDX43, STAG2, BRAF, CDKN2A, DUSP6, KRAS, RASA1, CIC, GNAQ, HRAS |
| Trastuzumab | Y | Y | N |  | Y | 1998 | Breast Cancer, Early Breast Cancer, Inflammatory Breast Cancer (IBC), Locally Advanced Breast Cancer (LABC), Metastatic Adenocarcinoma of the Gastro-Esophageal Junction, Metastatic Adenocarcinoma of the Stomach, Metastatic Breast Cancer, Metastatic Gastric Adenocarcinoma, Metastatic Gastroesophageal Junction Adenocarcinoma | ERBB2, FCGR2A, IFNG, FCGR3A, RNF8, BARD1, BIRC5, ERBB3, APC, PPCDC, PIK3CA, ARID1A, EPCAM, PTEN |
| Trastuzumab Deruxtecan | Y | Y | N |  | N | 2019 | HER2 Positive Breast Cancers | ERBB2 |
| Trastuzumab Emtansine | Y | Y | N |  | N | 2013 | Refractory, metastatic Non small cell lung cancer |  |
| Trastuzumab, Hyaluronidase | Y | Y | N |  | N | 1998 | Breast Cancer, Early Breast Cancer, Inflammatory Breast Cancer (IBC), Locally Advanced Breast Cancer (LABC), Metastatic Adenocarcinoma of the Gastro-Esophageal Junction, Metastatic Adenocarcinoma of the Stomach, Metastatic Breast Cancer, Metastatic Gastric Adenocarcinoma, Metastatic Gastroesophageal Junction Adenocarcinoma | ERBB2, FCGR2A, IFNG, FCGR3A, RNF8, BARD1, BIRC5, ERBB3, APC, PPCDC, PIK3CA, ARID1A, EPCAM, PTEN |
| Tretinoin | N | Y | Y |  | Y | 1971 | FAB classification M3 Acute promyelocytic leukemia | RXRB, RARG, RXRG, RARRES1, RORB, RARA, NR2C2, RARB, NR2E1, MYCN, FUS, CDKN1B, ALDH1A2, PML, BCOR, CYP26B1, NPM1, LCN1, P2RY2, GPRC5A, SMAD2, PDK4, RBP4, CYP26A1, CYP26C1, OBP2A, HPGDS |
| Trifluridine, Tipiracil | Y | Y | N |  | N | 1980 | Metastatic Colorectal Cancer (MCRC) | TYMS, SLC29A1 |
| Triptorelin | N | Y | Y |  | N | 2000 | Advanced Prostate Cancer | GNRHR, STS |
| Trofosfamide | N | N | Y |  | N |  | Non-Hodgkin lymphoma | CYP2B6 |
| Tucatinib | Y | Y | N |  | N | 2020 | Breast Cancer, Unresectable Breast Cancer | ERBB4, ERBB2, ERBB3 |
| Valrubicin | N | Y | N |  | N | 1998 | In situ BCG-refractory Bladder carcinoma | TOP2A, TOP2B |
| Vandetanib | Y | Y | N |  | N | 2011 | Metastatic Medullary Thyroid Cancer, Locally advanced Medullary thyroid cancer | EPHB1, EPHA4, RET, EPHB3, EPHA5, EPHA8, PTK6, EPHB2, EPHA1, EPHA10, EPHB6, EPHA3, EPHA7, EPHA6 |
| Vemurafenib | Y | Y | N |  | N | 2011 | Metastatic Melanoma, Unresectable Melanoma, Refractory Non-small cell lung cancer | BRAF, PREX2, CIC, STAG3, MAP2K1, STAG2, NRAS, NF1, ATXN1L, RAC1, SOX10 |
| Venetoclax | Y | Y | N |  | N | 2016 | Chronic Lymphocytic Leukaemia (CLL), Small Lymphocytic Lymphoma | BCL2L2, BCL2, BCL2L1, NPM1, IGH, IDH2 |
| Vinblastine | N | Y | Y |  | Y | 1965 | Advanced Soft Tissue Sarcoma, Cancer, Bladder, Kaposi&#39;s sarcoma, Lymphoma, Hodgkins, Metastatic Melanoma, Non-Small Cell Lung Carcinoma (NSCLC), Advanced Alibert-Bazin syndrome, Advanced Testicular cancer, Histiocytic lymphoma, Refractory Breast cancer | TUBB, TUBE1, TUBA1A, TUBD1, TUBG1, JUN, CGA, ABCB4, TRH, TUBB4A, PTHLH |
| Vincristine | N | Y | Y |  | Y | 1965 | Acute Lymphoblastic Leukaemia Recurrent, Acute Lymphocytic Leukemia (ALL), Choriocarcinoma, Chronic Lymphocytic Leukaemia (CLL), Ewing&#39;s Sarcoma, Hepatoblastomas, Kaposi&#39;s sarcoma, Lymphoma, Hodgkins, Multiple Myeloma (MM), Neuroblastomas, Non-Hodgkin&#39;s Lymphoma (NHL), Ovarian germ cell tumour, Pheochromocytomas, Retinoblastoma, Rhabdomyosarcomas, Small Cell Lung Cancer (SCLC), Wilms&#39; tumor, Advanced Thymoma | TUBA4A, CXCL12, LINC00251, ABCC10, CEP72, BMP7, ODC1, DROSHA, DOK5, PNPLA3, MYCN, GSTA1, ABCB4, RALBP1, ACTG1, ATRX, SRI, CAPG |
| Vincristine Liposome | N | Y | N |  | N | 2012 | Acute Lymphoblastic Leukaemia Recurrent, Acute Lymphocytic Leukemia (ALL), Choriocarcinoma, Chronic Lymphocytic Leukaemia (CLL), Ewing&#39;s Sarcoma, Hepatoblastomas, Kaposi&#39;s sarcoma, Lymphoma, Hodgkins, Multiple Myeloma (MM), Neuroblastomas, Non-Hodgkin&#39;s Lymphoma (NHL), Ovarian germ cell tumour, Pheochromocytomas, Retinoblastoma, Rhabdomyosarcomas, Small Cell Lung Cancer (SCLC), Wilms&#39; tumor, Advanced Thymoma | TUBA4A, CXCL12, LINC00251, ABCC10, CEP72, BMP7, ODC1, DROSHA, DOK5, PNPLA3, MYCN, GSTA1, ABCB4, RALBP1, ACTG1, ATRX, SRI, CAPG |
| Vindesine | N | N | Y |  | N | 1979 | Acute Lymphocytic Leukemia (ALL), Melanoma, Malignant | TUBB1, TUBB |
| Vinflunine | Y | N | N |  | N |  | Transitional Cell Carcinoma of the Urothelial Tract, Metastatic Transitional Cell Carcinoma of the Urothelial Tract | TUBB |
| Vinorelbine | N | Y | Y |  | Y | 1994 | Advanced Non Small Cell Lung Cancer, Esophageal Cancers, Locally Advanced Non-Small Cell Lung Cancer, Metastatic Breast Cancer, Recurrent Cervical Cancer, Soft Tissue Sarcoma (STS), Recurrent, IV-B Cervical cancer | TUBB, SMARCA4, CASP7, RRM1, APC, XRCC1, TUBB2A |
| Vismodegib | Y | Y | N |  | N | 2012 | Locally Advanced Basal Cell Carcinoma, Metastatic Basal cell carcinoma | PTCH1, SMO, SHH |
| Vorinostat | N | Y | N |  | N | 2006 | Persistent Cutaneous T-Cell Lymphoma, Progressive Cutaneous T-cell lymphoma, Recurrent Cutaneous T-cell lymphoma | HDAC8, HDAC3, HDAC7, HDAC1, HDAC4, HDAC11, HDAC5, HDAC2, HDAC10, HDAC6, HDAC9, SMARCB1, FBXW7, EZH2, MYD88, BAP1, CHD4 |
| Zanubrutinib | N | Y | N |  | N | 2019 | Mantle Cell Lymphoma (MCL) | BTK, BMX, TEC, BLK, ITK, ERBB4, PTK6, TXK, FGR, FRK |
| Relugolix | N | Y | N |  | N | 2020 | Advanced Prostate Cancer | GNRHR, LHCGR |
| Margetuximab | N | Y | N |  | N | 2020 | HER2-positive Metastatic Breast Cancer | ERBB2, ERBB3 |
| Naxitamab | N | Y | N |  | N | 2020 | High risk, refractory Neuroblastomas of the bone or bone marrow, High risk, relapsed Neuroblastomas of the bone or bone marrow |  |
